# A versatile fluorescent reporter for Usutu virus drug screening and infection tracking

**DOI:** 10.1093/pnasnexus/pgag328

**Published:** 2026-09-29

**Authors:** Amy N Nelson, Aaron E Lin, Saloni Sinha, Sydney J Mullin, Jianche Liu, Alexandra G Bell, Sebastian S Carver, Jason Sethiadi, Jiayu Zhang, Brigitte L Heller, Cameron Myhrvold, Robert E Schwartz, Alexander Ploss

**Affiliations:** Department of Molecular Biology, Princeton University, Princeton, NJ 08544, USA; Department of Molecular Biology, Princeton University, Princeton, NJ 08544, USA; Division of Gastroenterology and Hepatology, Department of Medicine, Weill Cornell Medicine, New York, NY 10065, USA; Department of Molecular Biology, Princeton University, Princeton, NJ 08544, USA; Department of Molecular Biology, Princeton University, Princeton, NJ 08544, USA; Computational and Systems Immunology Track, Stanford Immunology Program, Stanford University School of Medicine, Stanford, CA 94305, USA; Department of Molecular Biology, Princeton University, Princeton, NJ 08544, USA; Department of Molecular Biology, Princeton University, Princeton, NJ 08544, USA; Division of Gastroenterology and Hepatology, Department of Medicine, Weill Cornell Medicine, New York, NY 10065, USA; Department of Molecular Biology, Princeton University, Princeton, NJ 08544, USA; Department of Molecular Biology, Princeton University, Princeton, NJ 08544, USA; Department of Molecular Biology, Princeton University, Princeton, NJ 08544, USA; Omenn-Darling Bioengineering Institute, Princeton University, Princeton, NJ 08544, USA; Department of Chemical and Biological Engineering, Princeton University, Princeton, NJ 08544, USA; Department of Chemistry, Princeton University, Princeton, NJ 08544, USA; Division of Gastroenterology and Hepatology, Department of Medicine, Weill Cornell Medicine, New York, NY 10065, USA; Department of Molecular Biology, Princeton University, Princeton, NJ 08544, USA

**Keywords:** Usutu virus, USUV, orthoflavivirus, host responses, drug development

## Abstract

Usutu virus (USUV) is an emerging mosquito-borne orthoflavivirus closely related to West Nile and Japanese encephalitis viruses. Despite its increasing prevalence in Europe and reports of neuroinvasive disease in humans, the molecular and immunological determinants of USUV infection remain poorly understood, largely due to the absence of tractable experimental tools. Here, we report the generation and characterization of the first full-length fluorescent reporter USUV. Using a previously established infectious clone of the Vienna 2001 strain, we inserted the *mScarlet-I* gene into a permissive region of the viral genome, generating a stable and replication-competent reporter virus. In human hepatoma cells, USUV-mScarlet exhibited replication kinetics comparable to wild-type virus and maintained fluorescence over at least 10 serial passages. Application of this system identified remdesivir and interferon-alpha 2a (IFN-α2a) as potent USUV inhibitors and revealed robust induction of the type I IFN response in infected mouse fibroblasts, despite overall attenuation in this cell type. In IFN signaling deficient mice, which are permissive for viral replication, USUV-mScarlet infection resulted in transient disease and reporter expression in multiple peripheral tissues. Among splenocytes, specific myeloid cell subsets were highly enriched in reporter expression, suggesting their role as targets of viral infection. Collectively, these findings demonstrate that USUV-mScarlet recapitulates wild-type infection dynamics in human hepatoma cells while enabling single-cell/quantitative visualization and sorting of infected cells to assess viral spread and host responses. This reporter platform provides a versatile tool for studying USUV biology and will facilitate the development of targeted antivirals and vaccines against this emerging orthoflavivirus.

Significance statement This study establishes the first-reported full-length fluorescent reporter Usutu virus (USUV), enabling real-time visualization of infection dynamics in vitro and in vivo. The reporter virus retains near-wild-type replication in human hepatoma cells and reveals key interferon-mediated antiviral responses. Infection with this reporter virus in vivo results in milder clinical signs of disease compared to the nonreporter virus. Despite this attenuation, we can successfully identify cell subsets and detect viral RNA and proteins in peripheral tissues. Ultimately, USUV-mScarlet provides a powerful tool to study USUV pathogenesis that could accelerate antiviral and vaccine development.

## Introduction

Usutu virus (USUV) is an emerging mosquito-borne orthoflavivirus known for causing astounding lethality in Eurasian blackbirds (1, 2). This virus was originally isolated from mosquitoes in Eswatini in 1959 and has since circulated through African and European countries including Austria, Germany, Czechia, Poland, Belgium, Netherlands, Spain, Italy, Croatia, Greece, Uganda, Senegal, Central African Republic, and Burkina Faso (3–14). The transmission cycle includes *Culex pipiens* mosquito vectors and avian amplifying hosts (15–17). Notably, USUV has been detected in a variety of other zoonotic reservoirs, which do not support high levels of USUV replication, and are considered dead-end hosts. The vast majority of human infections have been asymptomatic; however, USUV has demonstrated a neuroinvasive capacity in immunocompromised individuals (11, 18–21). Neuroinvasive clinical signs have also been recorded in birds and mice (22–27). USUV is closely related to well-studied orthoflaviviruses including West Nile virus (WNV), Japanese encephalitis virus (JEV), and Zika virus (ZIKV), but has been the focus of fewer research studies due to its limited perceived threat to humans currently. Previous in vitro studies of USUV have investigated the susceptibility of cell lines or primary cells from a variety of species (26, 28–30), while previous in vivo studies have described disease progression in different mouse genotypes and the extent of neuroinvasive infection (25, 27, 31–34).

The development of reporter orthoflaviviruses has been pivotal for elucidating the spatiotemporal dynamics of infection and host–virus interactions. Fluorescent reporters, in particular, obviate the need for cell fixation and permeabilization, thereby facilitating flow cytometric analyses and live-cell confocal imaging. Such reporter viruses have proven invaluable and have facilitated studies of the cellular tropism of WNV (35), visualization of Langat virus spread in ex vivo tick organ culture models (36), and testing of ZIKV antiviral drug candidates (37).

A few distinct approaches have been utilized to generate orthoflavivirus reporter genomes, which are notoriously intolerant to the insertion of heterologous genes. For example, early constructs incorporated reporter genes into the 3′ untranslated regions (UTRs) of JEV or WNV genomes (35, 38, 39). However, these reporter viruses exhibited reduced replication efficiency relative to their wild-type counterparts, and serial passaging revealed instability of insertions at this site. Subsequent strategies placed reporter genes between the envelope (E) and nonstructural 1 (NS1) gene (40) or between the 5′ UTR and capsid (C) gene (41), often requiring duplication or modification of specific sequence elements to preserve correct proteolytic processing.

Currently, no reporter virus platforms exist for USUV, a limitation that hampers antiviral drug screening, real-time tracking of infection dynamics, and identification of USUV-harboring cell types in vivo. To address this gap, we constructed a full-length USUV reporter genome expressing the red fluorescent protein mScarlet. Building on reagents previously developed in our laboratory, we inserted the *mScarlet-I* gene into an infectious cDNA clone of the Vienna 2001 USUV strain (34), using an established cloning strategy (42–44). The resulting USUV-mScarlet reporter genome was stable over at least 10 serial passages and replicated with comparable kinetics to the wild-type clone in Huh7 human hepatoma cells. We applied USUV-mScarlet to characterize viral replication and host responses in vitro and in vivo.In cell culture, we identified interferon-alpha 2a (IFN-α2a) and remdesivir as potent inhibitors of USUV replication whereas other small molecule inhibitors of other orthoflaviviruses had minimal-to-no effect. We observed robust induction of type I IFN responses in infected mouse fibroblasts. In permissive mice with targeted disruptions in IFN signaling systems (Ifnar−/− and Stat1−/−), infection with USUV-mScarlet caused transient clinical signs with detectable reporter expression and viral antigen in multiple peripheral tissues. Notably, all animals recovered, indicating that the reporter virus is attenuated relative to its wild-type counterpart which is uniformly lethal at the same dose. To identify types of cells infected by USUV, we sorted for splenocytes enriched in mScarlet expression and found that myeloid cells were highly enriched by single-cell RNA sequencing, suggesting an important role for these cells as an early reservoir for USUV in vivo.

The immunological mechanisms governing USUV dissemination and persistence within a susceptible host remain largely unexplored, in part due to the lack of specific experimental tools. The establishment of a stable, replication-competent USUV reporter virus provides a versatile platform to dissect viral tropism, immune responses, and disease progression at cellular and organismal levels. Such tools will be instrumental for advancing the development of antivirals and vaccines against this emerging neurotropic orthoflavivirus.

## Results

### Generation and phenotypic characterization of a fluorescent USUV reporter genome

To enable real-time analysis of USUV infection dynamics, we generated a reporter virus expressing the fluorescent protein mScarlet-I (USUV-mScarlet). Our group previously constructed an infectious clone of the TC508 strain (USUV-WT), originally isolated from a blackbird in Austria in 2001, using the circular polymerase extension reaction (CPER) with eight overlapping viral fragments (34). To create the fluorescent reporter version, we inserted the *mScarlet-I* gene into a TOPO vector containing fragment 2 of the USUV genome (Fig. 1A). The insertion site and flanking sequence elements were adapted from prior luciferase- (42) and our mScarlet-based reporter designs for YFV-17D (43). For expression of a native viral proteolytic cleavage site, we duplicated the first nine amino acids of the NS1 protein—with altered codons to avoid homologous recombination—and inserted this upstream of mScarlet. Downstream of mScarlet, we added the transmembrane domain of envelope (E) derived from DENV-1 (DENV linker) (42) to facilitate correct polyprotein processing and reporter stability (40).

**Figure 1:**
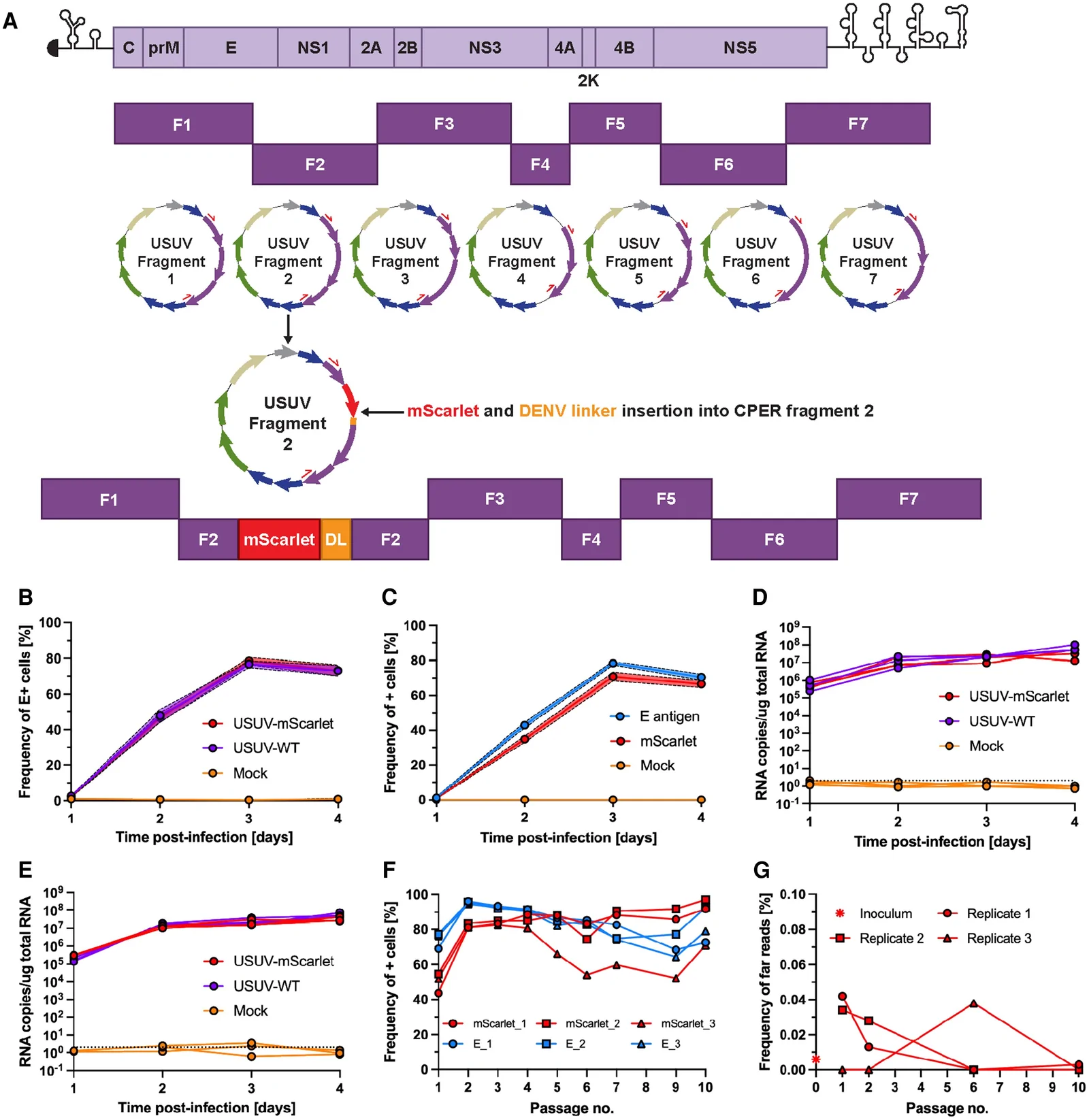
Generation and in vitro characterization of an mScarlet-expressing infectious clone of USUV TC508. A) mScarlet and a DENV linker sequence are inserted into the TOPO plasmid containing USUV fragment 2. An infectious clone is generated via the CPER as previously described. B) USUV-mScarlet has identical replication kinetics to the wild-type infectious clone in Huh7 cells 1–4 dpi (*n* = 3/day, shading indicates SEM). C) Viral envelope (E) staining slightly exceeds mScarlet signal in Huh7 cells 2–4 dpi with USUV-mScarlet (*n* = 3/day, shading indicates SEM). D) Huh7 cell lysates and E) cell culture supernatants contain highly comparable amounts of USUV-WT and USUV-mScarlet RNA 1–4 dpi (*n* = 3/day). The dotted line indicates the limit of detection. F) Comparison of E staining and mScarlet signal in Huh7 cells infected with USUV-mScarlet over 10 passages. Three samples were independently passaged: replicate 1 is shown with circles, replicate 2 is shown with squares, and replicate 3 is shown as triangles. G) Quantification of far bulk RNA sequencing reads, which indicates deletion of the mScarlet insertion, in the initial inoculum and cell culture supernatants from passages 1, 2, 6, and 10.

Following virus rescue, USUV-mScarlet yielded titers equivalent to USUV-WT as determined by focus-forming unit (FFU) assay. Infection of Huh7 human hepatoma cells at a multiplicity of infection (MOI) of 0.01 resulted in indistinguishable replication kinetics between the reporter and wild-type viruses from 1 to 4 days postinfection (dpi) (Fig. 1B), as judged by pan-orthoflavivirus E staining and flow cytometry. At 1 dpi, both viruses infected only 2–3% of cells. Infection expanded rapidly by 2 dpi (mean 47%) and peaked near 80% by 3 dpi. A slight decline to ∼73% on 4 dpi likely reflected cell death due to overgrowth or early cytopathic effects. To determine if mScarlet reporter expression accurately reflected viral replication, we compared direct fluorescence reporter detection against pan-orthoflavivirus E staining. In USUV-mScarlet-infected cells, the proportion of E antigen-positive cells consistently exceeded that of mScarlet-positive cells (Fig. 1C), most prominently at 2 dpi (43% E^+^ vs. 35% mScarlet^+^). This suggests that E staining is marginally more sensitive by flow cytometry than direct fluorescence detection. Nevertheless, intracellular and extracellular viral RNA levels were nearly identical between USUV-WT- and USUV-mScarlet-infected cells (Fig. 1D and E). These results collectively indicate that USUV-mScarlet retains wild-type replication dynamics and is phenotypically indistinguishable from USUV-WT in Huh7 cells.

To assess the genetic stability of the mScarlet insert, we serially passaged USUV-mScarlet in Huh7 cells for 10 consecutive rounds, quantifying the frequencies of E^+^ and mScarlet^+^ cells at each passage (Fig. 1F). As expected from the single-cycle analyses in Fig. 1C and E-positivity exceeded mScarlet-positivity during the first four passages. At passage 5, two replicates displayed slightly higher frequencies of mScarlet^+^ than E^+^ cells (88% vs. 85%). In contrast, the third replicate showed a marked decline in mScarlet-positivity (66%) despite maintaining similar levels of E-positivity (82%), suggesting a transient reduction in reporter expression. Across passages 6–9, mScarlet-positivity in this replicate remained lower (52–60%) but increased to 71% at passage 10. Notably, the frequencies of E^+^ cells gradually declined in all three replicates from passages 6–9, before rising again at passage 10.

To determine whether the reduction of reporter expression was associated with recombination-mediated excision of mScarlet from viral genomic RNA, we performed targeted RNA sequencing on the original inoculum and supernatants from passages 1, 2, 6, and 10. We reasoned that intact viral genomes should have mScarlet upstream of the start of the NS1 gene, while a large deletion of mScarlet would place the E gene in closer linear sequence proximity to NS1. Therefore, we devised a next-generation sequencing (NGS) library prep strategy to sequence cDNAs directly upstream of NS1, using gene-specific RT primers that anneal to nucleotides 40–58 of the NS1 gene and a Tn5 transposase akin to standard 3′ end sequencing methods. We quantified the frequency of NGS reads mapping an unexpectedly long distance from the primer (“far reads”), which would indicate deletion of the mScarlet region. Across all three biological replicates, the proportion of far reads was extremely low (≤0.042%), and the original inoculum showed only 0.006% far reads (Fig. 1G). These results demonstrate that no meaningful recombination events occurred during passaging, and that the mScarlet insert is stably maintained within the USUV genome for at least 10 passages.

### Remdesivir and IFN-α2a potently inhibit USUV infection

No antiviral therapies are currently approved for treatment of USUV infection in humans or other susceptible hosts. Several broadly acting compounds, including ribavirin (45), favipiravir (45, 46), the antiparasitic drug ivermectin (45–47), and the chemotherapeutic 5-fluorouracil (45), have been reported to exhibit partial anti-USUV activity; however, the dedicated evaluation of antiviral candidates, particularly those suitable for in vivo application, remains limited.

To demonstrate the utility of USUV-mScarlet in this context, we tested several compounds with known antiviral activity against other *Flaviviridae* members. Specifically, we evaluated the hepatitis C virus (HCV) therapeutics sofosbuvir (48) and ribavirin (49), the broad spectrum antiviral remdesivir (50); the DENV inhibitor JNJ-A07 which displays strong antiviral potency in both cultured cells and mouse models (51); and recombinant human interferon-α2a (IFN-α2a), a cytokine clinically used for chronic HCV infection and select malignancies. Human hepatoma (Huh7) cells were infected with USUV-mScarlet, then treated at 2 hours post-infection (hpi) with serial dilutions of each compound: 0.02–50 μM of sofosbuvir, ribavirin, remdesivir, or JNJ-A07 (3-fold dilutions), or 5.12 × 10^−5^–500 units of IFN-α2a (5-fold dilutions). In parallel, we quantified the cytotoxicity of each compound across the same concentration ranges.

With an IC_50_ of 2.82 μM, sofosbuvir began inhibiting USUV-mScarlet infection at concentrations ≥0.6 μM and achieved near-complete suppression at ≥16.7 μM (Fig. 2A). Ribavirin was less potent with an IC_50_ of 20.4 μM and detectable antiviral effects only at concentrations >5.6 μM (Fig. 2B). Remdesivir completely inhibited USUV-mScarlet infection at ≥1.8 μM and had an IC_50_ of 0.17 μM (Fig. 2C). JNJ-A07 showed minimal antiviral activity, reducing USUV-mScarlet spread only at the highest dose tested (Fig. 2D). In contrast, IFN-α2a exhibited robust antiviral activity with an IC_50_ of 0.075 units. Measurable inhibition began at ≥3.2 × 10^−2^ units and complete suppression of infection achieved at ≥0.8 units (Fig. 2E). Further, we investigated if IFN-α2a similarly inhibited USUV-WT infection and determined the IC_50_ to be 0.078 units (Fig. 2F). This concordance suggests that our reporter virus faithfully recapitulates wild-type virus drug responses.

**Figure 2:**
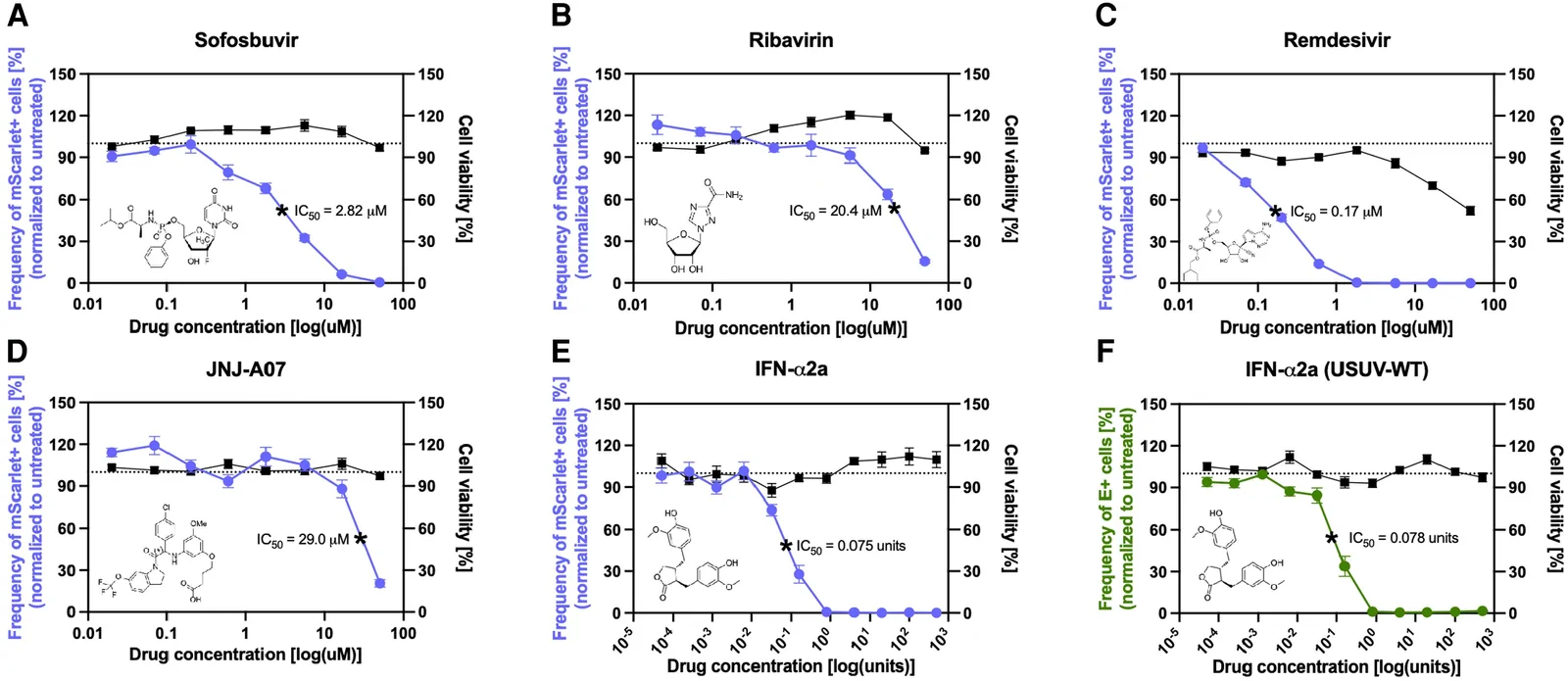
Evaluation of A) sofosbuvir, B) ribavirin, C) remdesivir, D) JNJ-A07, and E) IFN-ɑ2a for antiviral activity against USUV-mScarlet. F) Evaluation of IFN-ɑ2a for antiviral activity against USUV-WT. Tested concentrations of sofosbuvir, ribavirin, and JNJ A07 ranged from 0.02–50 µM. Tested concentrations of IFN-ɑ2a ranged from 5.12 × 10^−5^–500 units. For each drug concentration, the frequency of mScarlet^+^ cells is shown in periwinkle (*n* = 8/drug concentration), the frequency of E^+^ cells is shown in green, and cell viability is shown in black (*n* = 8/drug concentration). Frequency of mScarlet^+^ or E^+^ cells in the drug-treated conditions were normalized to the untreated condition and cell viability was normalized to the vehicle control (0.1% dimethyl sulfoxide [DMSO]). Asterisks indicate the IC_50_ values. Error bars indicate SEM. The dotted lines indicate 100% infection and 100% cell viability. The structural formula for each drug is shown in the bottom left corner of the graph.

High concentrations of remdesivir (≥16.7 μM) were cytotoxic to Huh7 cells as we observed 52 and 70% cell viability following treatment with 50 or 16.7 μM, respectively. None of the other compounds induced significant cytotoxicity at the tested concentrations, although high-dose (50 μM) treatment with sofosbuvir, ribavirin, or JNJ-A07 modestly reduced Huh7 cell proliferation. Overall, ribavirin and JNJ-A07 showed only limited activity and sofosbuvir exhibited moderate antiviral activity. We identified remdesivir and IFN-α2a as highly potent inhibitors of USUV-mScarlet infection in human hepatoma cells, highlighting the utility of our reporter for antiviral drug testing.

### Interferon signaling limits USUV-mScarlet spread in mouse fibroblasts

We previously established that USUV-mScarlet replicates with wild-type-like kinetics in Huh7 human hepatoma cells; however, Huh7 cells have blunted antiviral responses against multiple RNA viruses. To assess viral replication in other cell lines, we challenged L929 mouse fibroblasts, which we confirmed were permissive to USUV-WT in an earlier study (34). In L929 cells, the reporter virus displayed markedly impaired spread compared to the wild-type virus, which may be due to the type I IFN signaling competency of these cells (52, 53). Additionally, species-specific innate immune responses or differences in cell growth rates could contribute to varying abilities of Huh7 and L929 cells to sense and combat the reporter virus. Putatively, insertion of the mScarlet gene creates changes to the viral RNA structure that could elicit more robust antiviral responses in L929 cells compared to Huh7 cells, which could enable quicker identification of the virus and priming of nearby cells to resist infection. Additionally, the rapid proliferation rate of L929 cells could result in cell division before virus production has occurred.

We hypothesized that the attenuated spread of USUV-mScarlet might reflect heightened sensitivity to innate immune restriction. We therefore tested whether ruxolitinib, a JAK inhibitor that blocks IFN signaling (54), could enhance replication of the reporter virus. L929 cells were infected with USUV-mScarlet (MOI 0.01) and treated with 2 μM ruxolitinib replenished daily. In both treated and untreated cells, mScarlet-positivity remained <1% through 1–2 dpi. However, by 3 dpi, infection increased to 9% in ruxolitinib-treated cells compared to only 2% in untreated controls. This disparity increased substantially by 4 dpi: 55% of ruxolitinib-treated cells were mScarlet-positive, whereas only 5% of untreated cells were infected (Fig. 3A).

**Figure 3:**
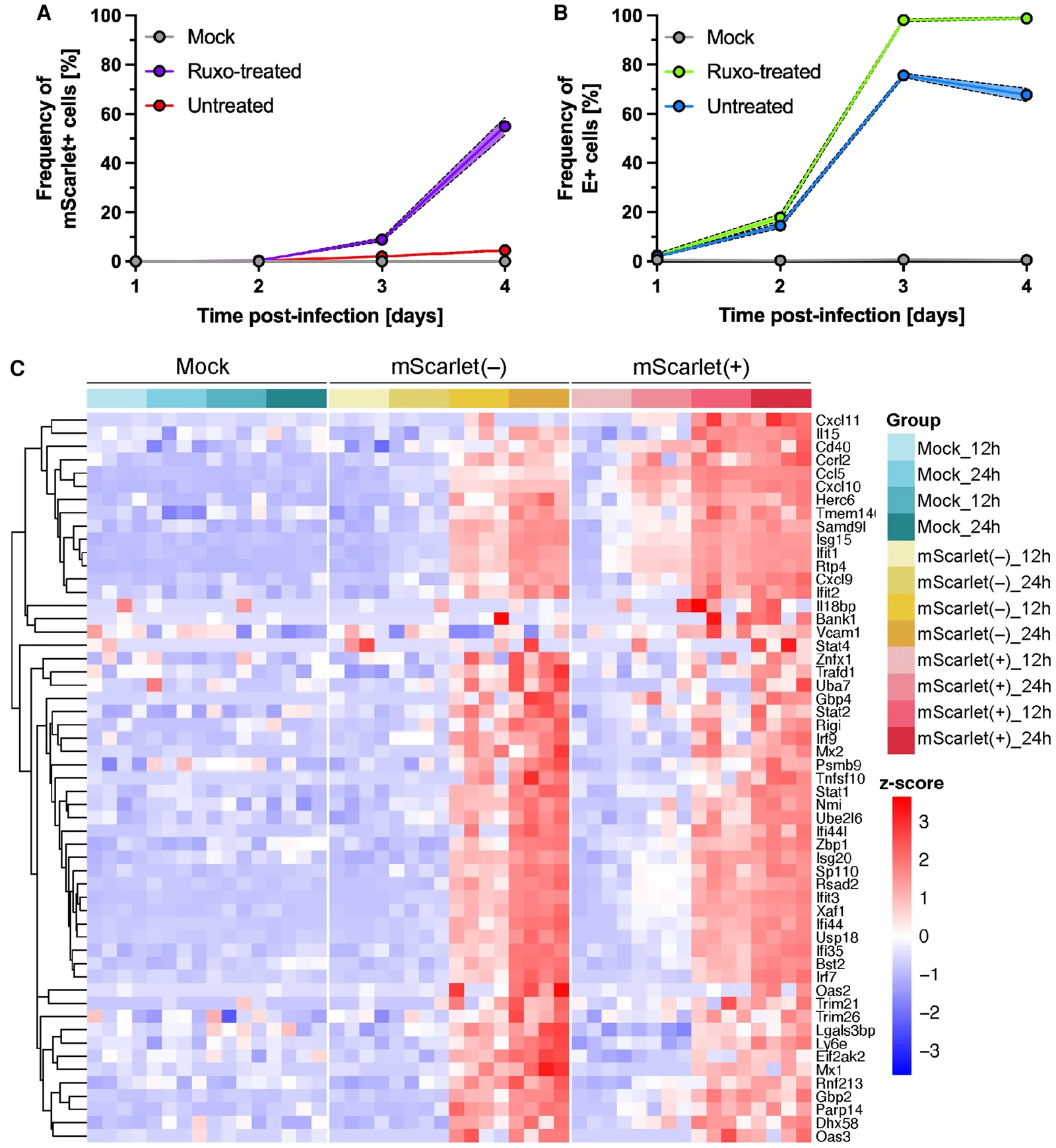
USUV-mScarlet infection leads to robust upregulation of ISGs in mouse fibroblasts. A) Replication kinetics of USUV-mScarlet (MOI: 0.01, *n* = 8/day) in L929 mouse fibroblasts 1–4 dpi with or without daily ruxolitinib treatment (2 µM). B) Replication kinetics of USUV-WT (MOI: 0.01, *n* = 8/day) in L929 mouse fibroblasts 1–4 dpi with or without daily ruxolitinib treatment (2 µM). Shading indicates SEM. C) A heatmap showing differential expression of 55 ISGs between the mock, mScarlet(−), and mScarlet(+) sorted cell populations at 12, 24, 36, and 48 hpi (*n* = 4/time point for each condition). These ISGs were associated with the HALLMARK_INTERFERON_ALPHA_RESPONSE or HALLMARK_INTERFERON_GAMMA_RESPONSE gene set published on the Molecular Gene Signatures Database and were significantly upregulated in mScarlet(−) and mScarlet(+) L929 cells at 48 hpi. Differential expression analyses were performed using DESeq2 to compare the mock vs. mScarlet(−) vs. mScarlet(+) cell samples at each time point. A gene was considered significantly differentially expressed if it had an adjusted *P*-value <0.05 and an absolute log2 fold change > 2. Heatmaps were generated with the pheatmap (v1.0.12) package, using the Z-score of each gene for visualization.

Further, we investigated whether ruxolitinib treatment could increase USUV-WT spread in L929 cells. Even without treatment, USUV-WT expanded efficiently in L929 cells, with 2% infection at 1 dpi, 15% at 2 dpi, a sharp increase to 76% on 3 dpi, and a slight drop to 68% on 4 dpi due to overconfluency. With ruxolitinib treatment, infection levels were highly comparable to the untreated group 1–2 dpi; however, total infection (> 99%) was achieved by 3 dpi and maintained at 4 dpi (Fig. 3B).

Together, these results indicate that the reduced spread of USUV-mScarlet in mouse fibroblasts is largely driven by IFN-mediated restriction that can be effectively rescued by pharmacologic inhibition of JAK-STAT signaling. Additionally, complete USUV-WT infection in L929 cells could only be achieved with ruxolitinib treatment, indicating that this virus is also IFN-sensitive.

### USUV-mScarlet induces a strong IFN-stimulated gene response in L929 mouse fibroblasts within 36 hpi

The host transcriptional response to USUV infection remains poorly defined, in part because traditional approaches for isolating infected cells rely on intracellular antigen staining, which requires fixation and permeabilization that can compromise RNA integrity. The fluorescent nature of USUV-mScarlet overcomes this limitation by enabling the direct isolation of infected, reporter-positive cells by fluorescence-activated cell sorting (FACS) while preserving intact cells and therefore high-quality RNA for sequencing. The gating scheme for collecting mScarlet-positive and mScarlet-negative cells from the same virally inoculated sample can be found in the Fig. S1A. We profiled the transcriptomic response to USUV-mScarlet in mScarlet-positive or negative L929 mouse fibroblasts at 12, 24, 36, and 48 hours postinfection (hpi), with a focus on interferon-stimulated genes (ISGs) (Fig. 3C). Genes with an adjusted *P*-value <0.05 and an absolute log_2_ fold change > 2 were considered significantly differentially expressed (Fig. S1B).

At 12 hpi, only four ISGs were significantly upregulated in mScarlet-positive cells—*Cxcl10*, *Rtp4*, *Ifit1*, and *Isg15*—indicating an early but limited antiviral response. These genes encode well-characterized antiviral and immunoregulatory proteins, including the chemokine Cxcl10, which promotes immune cell recruitment (55–57), and the antiviral effectors Rtp4, Ifit1, and Isg15, each of which restricts viral infection through distinct mechanisms (58–60). The transcriptional response expanded substantially over time: 19 ISGs were significantly upregulated at 24 hpi, 39 at 36 hpi, and 50 at 48 hpi. The four early-induced genes remained significantly upregulated throughout the time course. All ISGs detected at 24 hpi were also upregulated at 36 hpi, and all except *Irf1* remained upregulated at 48 hpi, indicating a steadily intensifying IFN response. Several ISGs, including *Ifi44* and *Rsad2* (viperin), as well as the chemokine *Cxcl11* and the chemokine receptor *Ccrl2*, showed monotonically increasing expression across the final three time points. *Ifi44* and *Rsad2* are canonical type I interferon-induced antiviral genes (61, 62), whereas *Cxcl11* and *Ccrl2* respond to type I and type II IFNs (63–65). ISGylation-associated factors such as *Herc6* and *Usp18* (66, 67) were also significantly upregulated from 24 to 48 hpi. Notably, *Mx2* and *Ifi44l* were among the most strongly induced ISGs at 36 and 48 hpi, showing log_2_ fold changes exceeding 7 and 9, respectively. Complete ISG lists for each time point are provided in Dataset S1.

The mScarlet-negative population may include uninfected cells as well as lowly infected cells that were capable of preventing high levels of viral replication. Viral RNA was detected in the mScarlet-negative group by bulk RNAseq and qPCR analyses, but at significantly lower levels compared to the mScarlet-positive subset (Fig. S2A and B). The mScarlet-negative cell population displayed a slightly delayed but ultimately broad antiviral transcriptional response (Fig. 3C). At 12 hpi, only *Vcam1* was significantly upregulated, and at 24 hpi only *Rtp4* met significance thresholds. By 36 hpi, however, 29 ISGs were significantly upregulated, increasing to 47 ISGs at 48 hpi. Several ISGs, including *Ifit2*, *Irf7*, *Irf9*, *Isg20*, *Mx1*, *Mx2*, *Oas3*, *Parp14*, *Rnf213*, and *Stat1*, were significantly upregulated in mScarlet-negative cells at 36 and 48 hpi but were not significantly induced in mScarlet-positive cells. Other ISGs, such as *Ly6e*, *Nmi*, *Oas2*, *Psmb9*, *Trafd1*, *Uba7*, and *Ube2l6*, were shared between mScarlet-positive and negative populations at later time points. The four ISGs that were consistently upregulated in mScarlet-positive cells at all time points—*Cxcl10*, *Rtp4*, *Ifit1*, and *Isg15*—were also significantly upregulated in mScarlet-negative cells, but only at 36 and 48 hpi, consistent with secondary induction through paracrine interferon signaling.

Gene set enrichment analysis revealed that the transcriptional responses in both mScarlet-positive and negative cells aligned strongly with the IFN alpha and gamma signatures (Fig. S3A and B). Overlap between significantly induced ISGs in these two cell populations at each time point is shown in Fig. S4A–D. Collectively, these data demonstrate that USUV-mScarlet induces a robust and steadily expanding ISG response in L929 fibroblasts, with most ISGs strongly upregulated by 36–48 hpi in both mScarlet-positive and negative populations.

### USUV-mScarlet is attenuated in vivo but establishes systemic infection in Ifnar−/− and Stat1−/− mice

We previously observed that USUV-WT was lethal to immunodeficient mice (34), and wanted to assess whether USUV-mScarlet would be similarly pathogenic. We challenged Ifnar−/− or Stat1−/− mice with 10^3^ FFU of USUV-WT or USUV-mScarlet. Consistent with our earlier findings, USUV-WT resulted in uniform lethality by 6 dpi. In contrast, USUV-mScarlet displayed marked attenuation. Seventy-five percent (3 of 4) of Ifnar−/− mice survived infection with 10^3^ FFU of USUV-mScarlet and fully recovered by 11 dpi, although all animals exhibited transient clinical signs from 4 to 6 dpi, including ruffled fur, reduced mobility, and difficulty being scruffed (Fig. 4A–C). Escalating the inoculum of USUV-mScarlet did not result in uniform mortality: Ifnar−/− mice challenged with 10^4^ FFU survived uniformly, whereas those receiving 10^5^ or 10^6^ FFU showed 50 and 75% survival, respectively (Fig. 4D). Transient weight loss occurred at all doses and was most severe at 10^5^–10^6^ FFU (Fig. 4E and F).

**Figure 4:**
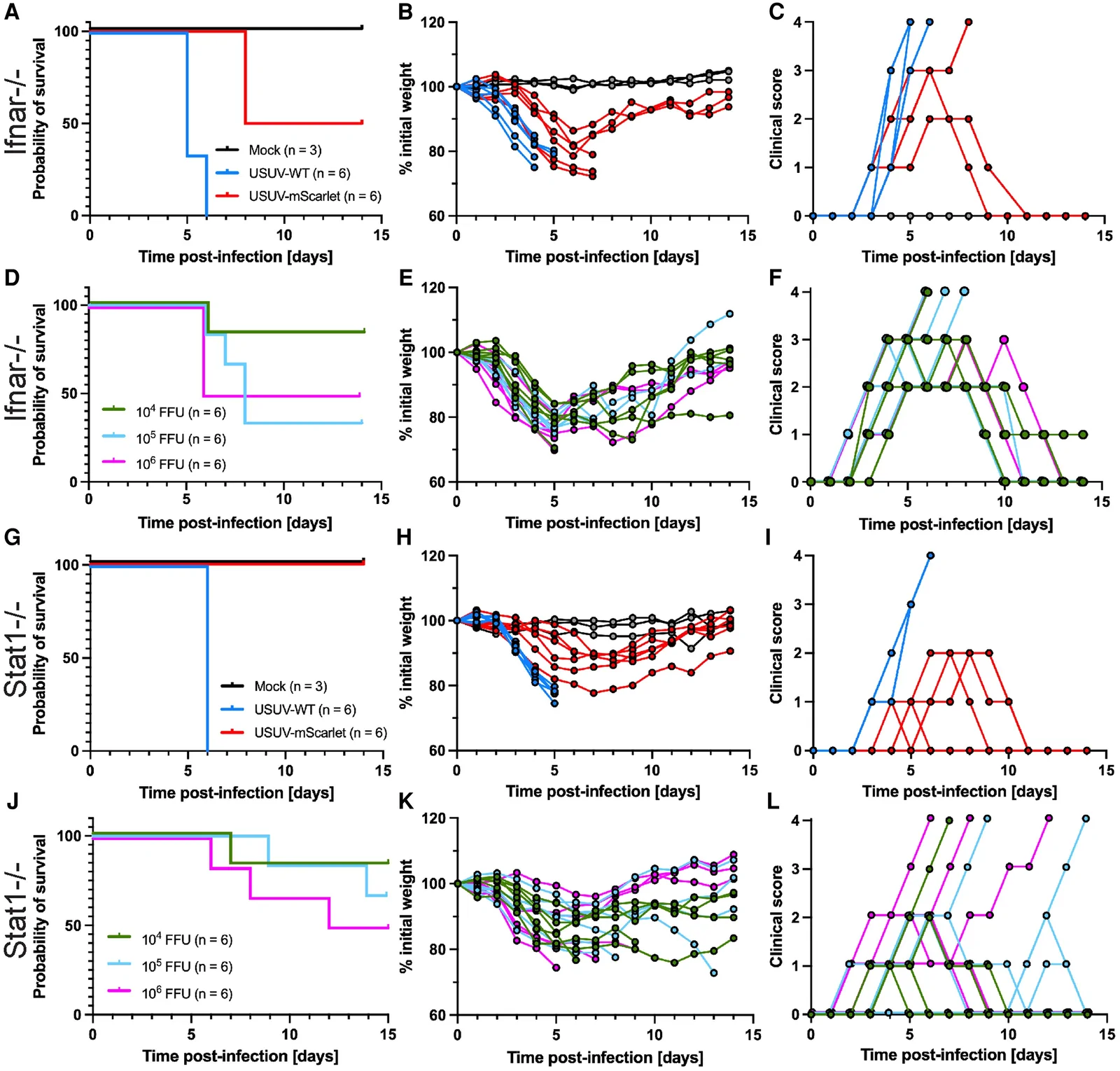
USUV-mScarlet infection is less pathogenic in Ifnar−/− and Stat1−/− mice compared to USUV-WT infection. A) Survival, B) weight changes, and C) clinical scoring of Ifnar−/− mice following infection with 10^3^ FFU of USUV-WT (*n* = 6) or USUV-mScarlet (*n* = 6) compared to mock mice (*n* = 3). D) Survival, E) weight changes, and F) clinical scoring of Ifnar−/− mice following infection with 10^4^–10^6^ FFU of USUV-mScarlet (*n* = 6/dose). G) Survival, H) weight changes, and I) clinical scoring of Stat1−/− mice following infection with 10^3^ FFU of USUV-WT (*n* = 6) or USUV-mScarlet (*n* = 6) compared to mock mice (*n* = 3). J) Survival, K) weight changes, and L) clinical scoring of Stat1−/− mice following infection with 10^4^–10^6^ FFU of USUV-mScarlet (*n* = 6/dose).

A similar attenuation phenotype was observed in Stat1−/− mice. Infection with 10^3^ FFU of USUV-mScarlet resulted in 100% survival, in stark contrast to the uniform lethality caused by USUV-WT at the same dose (Fig. 4G). Stat1−/− mice exhibited moderate clinical illness and transient weight loss between 4 and 7 dpi, but overall disease severity was reduced compared to Ifnar−/− mice (Fig. 4H and I). At 6 dpi, Ifnar−/− mice averaged 82.1% of their starting weight, whereas Stat1−/− mice retained 90.4%; by 7 dpi, these values were 84.4 and 88.4%, respectively. For Stat1−/− mice, increasing the inoculum of USUV-mScarlet to 10^4^–10^6^ FFU did not produce consistent lethality. Only one mouse infected with 10^5^ FFU succumbed to lethality on day 14, after partially recovering between 9 and 10 dpi then deteriorating again after 11 dpi. Together, these data show that USUV-mScarlet causes signs of acute infection in immunodeficient mice, but is ultimately controlled and is therefore attenuated compared to USUV-WT, which causes lethality.

### USUV-mScarlet establishes systemic infection with pronounced liver and spleen pathology in Ifnar−/− mice

To characterize tissue tropism and pathology in vivo, we inoculated Ifnar−/− mice with 10^3^ FFU of USUV-mScarlet and collected tissues at 6 dpi when clinical signs were most severe. Histopathological analysis revealed extensive hepatic necrosis with large foci of hepatocyte death and marked disruption of splenic architecture accompanied by dense immune cell infiltration (Fig. 5A). By contrast, we did not observe major pathological alterations in kidney or lung, nor significant neuronal loss or gliosis in the brain (Fig. S5). These findings closely paralleled our previously reported lesions in USUV-WT-infected mice (34).

**Figure 5:**
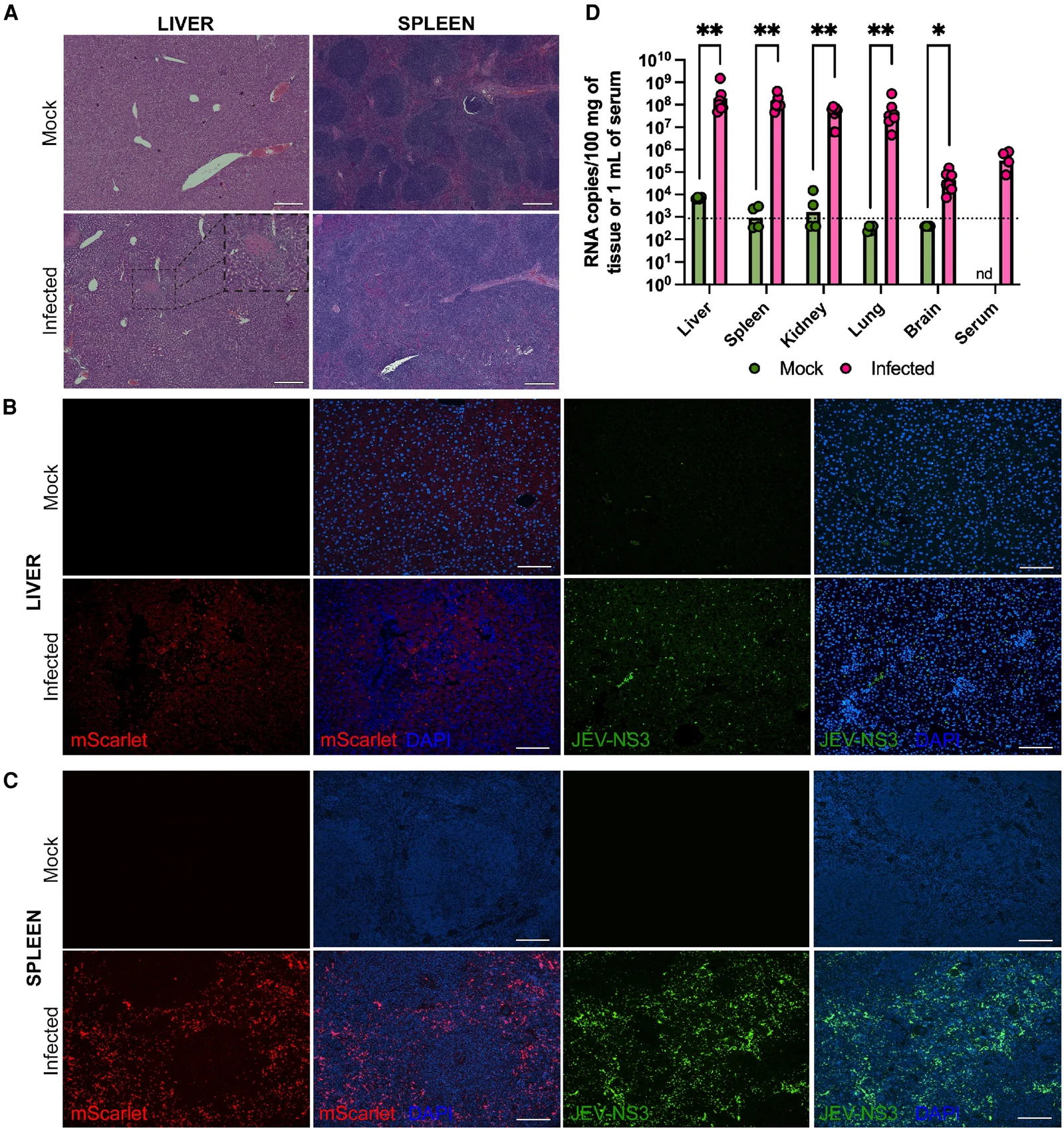
USUV-mScarlet RNA and proteins are detected in peripheral organs at 6 dpi. A) H&E staining of spleen and liver sections harvested from mock or USUV-mScarlet-infected mice. Infected spleens exhibited marked immune cell infiltration and disruption of tissue architecture. Infected livers showed large necrotic foci with widespread hepatocyte death. Scale bars indicate 200 µm. Immunostaining of B) liver and C) spleen tissue sections for mScarlet and JEV NS3 proteins confirmed robust reporter expression in and viral spread to infected tissues. Nuclei were counterstained with DAPI. Scale bars indicate 200 µm. D) Quantification of viral RNA in liver, spleen, kidney, lung, and brain tissue sections as well as serum harvested from Ifnar−/− mice (*n* = 2 for mock, *n* = 4 for infected) at 6 dpi. nd, not detected. The dotted line indicates the lower limit of quantification. Infected mice were subcutaneously inoculated with 10^3^ FFU of USUV-mScarlet. From each mouse tissue, two sections were prepared for qPCR analysis.

Immunostaining for the viral NS3 protein confirmed robust hepatic infection and revealed infection disseminated to the spleen, kidney, lung, and, to a lesser extent, the brain (Figs. 5B and C and S6A). In the brain, infected cells were sparse and largely confined to isolated cortical or perivascular regions. The kidney showed mild interstitial alterations with numerous infected tubular and interstitial cells, while the lung displayed diffuse mScarlet^+^/NS3^+^ staining and mild alveolar septal thickening. Quantification of infected cells showed no difference between mScarlet and NS3 staining in the liver, spleen, or kidney, although NS3 staining exceeded mScarlet staining in the lung and brain (Fig. S6B). Next, we wanted to quantify the extent of viral replication within each tissue and found viral RNA to be widely disseminated. We detected the highest viral loads in the liver, spleen, kidney, and lung (Fig. 5D).

Collectively, these data demonstrate that USUV-mScarlet establishes a systemic infection in Ifnar−/− mice with the most substantial viral burden and pathology in the liver and spleen, while maintaining detectable replication in multiple distal organs. Although viral dissemination largely mirrors that of USUV-WT (34), the markedly increased survival of Ifnar−/− and Stat1−/− mice infected with USUV-mScarlet underscores the attenuation of the reporter virus in vivo. This attenuation, combined with preserved viral spread and antigen detectability across tissues, highlights the utility of USUV-mScarlet as a tool for dissecting in vivo infection dynamics.

### Identification of mScarlet-positive immune cells in infected mouse splenocytes enables in vivo tracking of USUV infection

Beyond detecting viral RNA and protein, we next evaluated whether USUV-mScarlet could be used to identify which immune cell subsets were infected in vivo. We focused this initial analysis on hematopoietic cells as these were previously shown to be important reservoirs for orthoflaviviruses (68–70). Wild-type C57BL/6 mice carrying Stat1 floxed alleles (Statfl/fl, (71)) and Stat1−/− mice were inoculated with USUV-mScarlet. Initial flow cytometric analysis of splenocytes harvested at 3–4 dpi revealed a small but distinct population of CD45^+^ mScarlet^+^ cells in Stat1−/−mice, whereas even fewer fluorescent cells were detected in Stat1−/− controls (data not shown). In conjunction with detection of mScarlet, JEV NS3, and viral RNA in spleen sections (Fig. 5B–D), these findings suggest that, in the absence of STAT1-mediated signaling, the reporter virus could replicate to detectable levels in immune cells.

To define the immune cell types harboring the virus, we performed single-cell RNA sequencing (scRNA-seq) on FACS-sorted splenocytes from both genotypes at 3 dpi (gating scheme available in Fig. S7). We analyzed four populations: CD45^+^ splenocytes from an uninfected (mock) Stat1fl/fl mouse, CD45^+^ splenocytes from an infected Stat1fl/fl mouse, and CD45^+^ mScarlet^−^ and CD45^+^ mScarlet^+^ splenocytes from an infected Stat1−/− mouse. Because mScarlet^+^ cells were not detected in Stat1fl/fl animals, no equivalent fraction could be isolated from that genotype. Following scRNA-seq, we clustered cell transcriptomes from all four samples, identifying 28 total clusters (Table S4). Using canonical lineage markers (Fig. S8), these clusters grouped into three major cell classes: myeloid (*Itgam/CD11b* and *Ifitm2*), B (*Ebf1*), and T (*Cd3d*) lineages (Fig. 6A–D).

**Figure 6:**
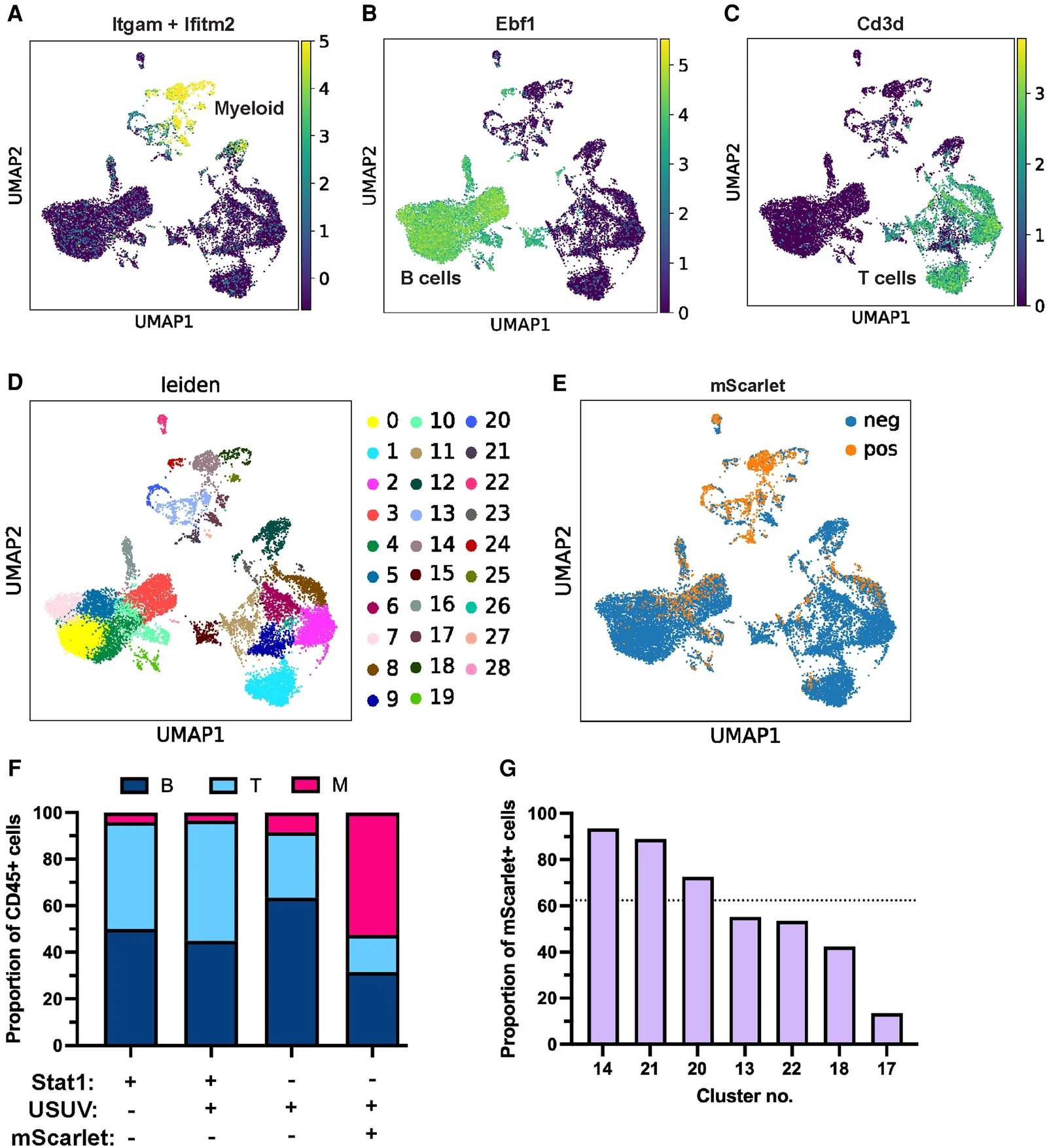
Specific myeloid cell subsets harboring USUV-mScarlet were identified by scRNA-seq of mouse splenocytes at 3 dpi. Four splenocyte samples were collected by FACS and subjected to scRNA-seq: CD45^+^ cells from a mock Stat1fl/fl mouse, CD45^+^ cells from an infected Stat1fl/fl mouse, CD45^+^ mScarlet^−^ cells from and infected Stat1−/− mouse, and CD45^+^ mScarlet^+^ cells from an infected Stat1−/− mouse. Cell types were grouped into three major clusters. A) Myeloid cell cluster identified by *Itgam* (*Cd11b*) and *Ifitm2* transcript coexpression. B) B lymphocyte cluster identified by *Ebf1* transcript expression. C) T lymphocyte cluster identified by *Cd3d* transcript expression. D) UMAP plot visualizing the subclustering of cells into 28 groups using the Leiden algorithm. The identity of each cluster is listed in Table S4. E) UMAP plot visualizing the distributions of mScarlet^−^ (blue) and mScarlet^+^ (orange) splenocytes across all clusters. F) Proportions of B cells, T cells, and myeloid (M) cells relative to all CD45^+^ splenocytes in each sample. Rows below the bar graph indicate mouse identity, infection status, and mScarlet expression status. Mouse identity is indicated as + for Stat1fl/fl and—for Stat1−/−. Infected mice are indicated as + and mock mice are indicated as −. mScarlet^+^ cell are indicated as + and mScarlet^−^ cells are indicated as −. G) Proportion of mScarlet^+^ cells relative to total CD45^+^ cells in all myeloid-lineage clusters containing over 100 cells. The dotted line indicates the proportion of mScarlet^+^ cells relative to total myeloid cells.

Mapping mScarlet^+^ and mScarlet^−^ cells across clusters revealed a clear separation of infected and uninfected immune compartments. The mScarlet^−^ population was broadly distributed with highest frequency among B and T cell clusters, whereas mScarlet^+^ cells localized almost exclusively to myeloid clusters (Fig. 6E). Quantifying this formally, myeloid cells comprised <5% of CD45^+^ splenocytes from uninfected or USUV-challenged Stat1fl/fl mice (Fig. 6F), which were not permissive to USUV infection in our previous study (34). Challenging Stat1−/− mice, which are permissive, with USUV-mScarlet led to a modestly higher frequency of myeloid cells (9%) among splenocytes negative for mScarlet (CD45^+^ mScarlet^−^, ∼99.8% of all splenocytes). Most strikingly, sorting on mScarlet signal (CD45^+^ mScarlet^+^ splenocytes) resulted in >50% myeloid cells, a 6-fold enrichment compared to CD45^+^ mScarlet^−^ splenocytes. This observation that myeloid cells are the major cell target of USUV-mScarlet infection in vivo is consistent with markers of viral infection being primarily excluded from the B cell- and T cell-rich white pulp region of the spleen (Fig. 5C) and is further consistent with our previous observation that mice deficient of B, T, and NK cells are not permissive to pathogenic USUV-WT infection in vivo (34).

Elucidating the immune cell compositions of each splenocyte sample confirmed that CD45^+^ mScarlet^+^ splenocytes from infected Stat1−/− mice exhibited a marked expansion of myeloid-lineage cells with a corresponding decrease in B and T cell proportions (Fig. 6F). In contrast, CD45^+^ splenocytes from both mock and infected Stat1fl/fl mice contained comparable proportions of B and T cells, with myeloid cells comprising <5% of the population.

To pinpoint which myeloid subsets were permissive to USUV-mScarlet, we compared the frequencies of mScarlet^+^ and mScarlet^−^ cells in clusters containing more than 100 cells (clusters 13, 14, 17, 18, 20, 21, and 22). Whereas 62% of all myeloid cells were mScarlet^+^, three clusters exhibited particularly high mScarlet^+^ frequencies, and we inferred their cell type identities based on their top 50 differentially expressed transcripts (Dataset S2): cluster 14 (early inflammatory monocytes: 94%), cluster 21 (spleen-resident red-pulp macrophages: 89%), and cluster 20 (migratory conventional dendritic cells: 72.5%) (Fig. 6G). Collectively, these data establish that USUV-mScarlet serves as an effective in vivo reporter for identifying infected immune cell subsets and tracking viral dissemination within the host.

## Discussion

In this study, we generated and characterized a full-length infectious fluorescent reporter USUV clone, USUV-mScarlet, and demonstrated its broad utility for antiviral screening, transcriptomic analyses, and in vivo infection tracking. The mScarlet insertion was highly stable across serial passages and did not impair viral replication in human hepatoma cells. Compared to wild-type USUV, the reporter virus exhibited reduced replication in murine fibroblasts and markedly attenuated pathogenicity in Ifnar−/− and Stat1−/− mice. Despite this attenuation, USUV-mScarlet established systemic infection and enabled the visualization and isolation of infected immune cell subsets in vivo. Single-cell transcriptomic analysis revealed an enrichment of mScarlet-positive cells within distinct myeloid populations, particularly inflammatory monocytes, migratory dendritic cells, and splenic macrophages, which conceivably contribute to early viral dissemination.

To our knowledge, USUV-mScarlet represents the first full-length fluorescent reporter system for USUV. Its modular design, generated through CPER, provides a versatile framework for inserting heterologous sequences into orthoflavivirus genomes. This flexibility facilitates future adaptation of the reporter backbone for additional fluorophores or functional elements, thereby expanding the currently limited toolkit available for studying USUV biology. The in vivo applications of USUV-mScarlet are, however, presently endpoint-restricted, as tissue processing for microscopy, flow cytometry, or qPCR requires terminal sampling. Complementary luciferase-based or recombinase-expressing reporter clones could enable longitudinal studies of infection dynamics and cellular tropism. For example, luciferase reporters permit real-time, noninvasive monitoring of viral spread by in vivo imaging, as demonstrated for dengue virus (72), while recombinase-based reporters allow indelible labeling of cells that have undergone viral entry, irrespective of productive replication (73). The future generation of analogous USUV reporters would greatly expand experimental capacity to dissect early events in viral dissemination and tissue tropism.

The stability of the mScarlet insertion observed here is notable. Reporter genes placed between the structural and nonstructural regions of orthoflavivirus genomes, such as in YFV-17D constructs expressing Gaussia luciferase or mScarlet, have shown exceptional genetic stability (42, 43). In contrast, earlier reporter constructs engineered into the 3′ untranslated region were prone to rapid loss of the insert, often within a few replication cycles (35, 39). Across 10 serial passages, USUV-mScarlet maintained robust reporter expression in three independent replicates, underscoring that this insertion site confers sustained stability without compromising replication fitness in vitro.

The IFN response emerged as a dominant determinant of USUV restriction in our study. We previously showed that intact IFN signaling in hematopoietic cells is critical for controlling USUV-WT infection in vivo (34). Here, our USUV-mScarlet reporter virus triggered strong induction of ISGs in L929 cells, which was driven by IFN alpha and gamma signaling. Consistently, pharmacologic blockade of JAK-STAT signaling enhanced reporter virus replication in mouse fibroblasts, while treatment with exogenous IFN-α2a potently inhibited viral spread in human hepatoma cells. Therefore, it is not surprising that this reporter virus was also significantly attenuated compared to USUV in vivo, and in fact suggests that, as with all viruses, USUV employs mechanisms to counter host antiviral defenses while USUV-mScarlet might be slightly less efficient in such measures. Because USUV infects and causes mortality in avian species, future studies will aim to delineate species-specific differences in antiviral signaling and the mechanisms by which USUV counteracts these responses in birds and mammals.

Although USUV-mScarlet is significantly attenuated in vivo, it remains a powerful experimental platform. The reporter allowed identification of specific immune cell subsets that harbor virus early during infection, suggesting potential reservoirs that may sustain replication or mediate systemic spread. Its attenuation somewhat constrains the interpretation of global transcriptomic responses, as the magnitude of viral replication in vivo likely differs from that of wild-type USUV. Future work may explore alternative genomic insertion sites or regulatory elements to partially restore wild-type pathogenicity while preserving fluorescence, thereby extending the utility of a reporter virus for later infection stages.

Together, our findings establish USUV-mScarlet as a genetically stable and experimentally tractable tool for dissecting USUV biology. The system enables real-time monitoring of infection, facilitates high-resolution studies of host immune responses, and provides a foundation for developing complementary reporter platforms. Insights gained from such tools will be critical for understanding the cellular and molecular determinants of USUV pathogenesis and for informing rational vaccine and therapeutic strategies. As orthoflaviviruses continue to expand geographically with a changing climate, advancing such molecular tools is essential to improve preparedness for emerging arboviral threats.

## Materials and methods

### Ethics statement

All animal experiments described in this study were performed in accordance with protocols that were reviewed and approved by the Institutional Animal Care and Use and Committee of Princeton University (number 3063). All mice were maintained in facilities accredited by the Association for the Assessment and Accreditation of Laboratory Animal Care (AAALAC). All work with infectious USUV was reviewed and approved by the Institutional Biosafety Committee of Princeton University (protocol registration number 1145).

### Cell lines and antibodies

Huh7 (RRID:CVCL_0336) and Huh7.5 (RRID:CVCL_7927) cells were kindly provided by Dr Charles Rice at The Rockefeller University. The L929 (CCL-1) cell line was obtained from the American Type Culture Collection (ATCC, Manassas, VA). Cells were maintained in Dulbecco's Modified Eagle Medium (DMEM) with high glucose and pyruvate (Thermo Fisher Scientific, Waltham, MA) supplemented with 10% (vol/vol) fetal bovine serum (FBS) and 1% (vol/vol) P/S. Cells were incubated at 37 °C with 5% CO_2_. Once confluent, cells were washed with Dulbecco's Phosphate-Buffered Saline (DPBS). Huh7 and Huh7.5 cells were treated with 0.05% trypsin-ethylenediaminetetraacetic acid (EDTA) in Hanks’ Balanced Salt Solution while L929 cells were treated with 0.25% trypsin-EDTA in Hanks’ Balanced Salt Solution, then replated. All antibodies used in this study are listed below in Table S1.

### Plasmids and virus production

To create the CPER fragment 2 reporter plasmid, a three-piece overlapping extension PCR was performed to insert the duplicated NS1 element, the *mScarlet-I* gene, and a DENV-1 E linker sequence into the wild-type CPER fragment 2 plasmid using the primers and templates listed in Table S2. Insertion of these sequences was based on our previous generation of a YFV-17D-mScarlet reporter (43, 44). The duplicated element contains the N-terminal 27 nucleotides of the NS1 gene with synonymous mutations introduced to prevent recombination with the native NS1 gene. Equimolar concentrations of the three fragments were used for ligation by overlapping extension PCR using the CloneAmp HiFi PCR premix (Takara Bio USA, Inc, San Jose, CA). The following thermocycler method was used: 98 °C for 1 min; 8 cycles of 98 °C for 10 s, 52 °C for 15 s, 72 °C for 30 s; 72 °C for 1 min. The primers PU-O-11167 and PU-O-11172 were used to select for amplicons containing all three fragments. The following thermocycler method was used: 98 °C for 1 min; 18 cycles of 98 °C for 10 s, 65 °C for 15 s, 72 °C for 50 s; 72 °C for 1 min. To generate the vector backbone, the original CPER fragment 2 plasmid was digested with AvrII and AleI-v2 (New England Biolabs Inc., Ipswich, MA) for 1 h at 37 °C. The overlapping extension PCR product was ligated to the digested backbone using T4 ligase (Thermo Fisher Scientific, Waltham, MA). Both viruses were titrated by FFU assay on Huh7.5 cells as previously described (34).

### Replication kinetics via flow cytometry

Huh7 cells were seeded in a 48-well plate at a density of 9.6 × 104 cells/well (80% confluency). L929 cells were seeded in a 48-well plate at a density of 7.2 × 104 cells/well (60% confluency). Virus inoculation was performed with the indicated MOI for 2 h at 37 °C. Following incubation, the inoculum was aspirated and replaced with 0.5 mL/well of DMEM containing high glucose and pyruvate + 10% (vol/vol) FBS + 1% (vol/vol) P/S. For ruxolitinib treatment of L929 cells, ruxolitinib (Selleck Chemicals, Houston, TX) was diluted in DMEM containing high glucose and pyruvate supplemented with 10% (vol/vol) FBS and 1% (vol/vol) P/S to a concentration of 2 µM and added at 0.5 mL/well. Ruxolitinib medium was replenished daily. To characterize viral replication kinetics, cells were collected on 1–4 days postinfection (dpi). All samples were collected in quadruplicate. On collection days, cells were washed with DPBS, treated with 0.05% (vol/vol) trypsin in Hank's Balanced Salt Solution (Thermo Fisher Scientific), then quenched with supplemented cell culture medium. Following a wash with DPBS, cells were fixed using 4% (vol/vol) paraformaldehyde (PFA). Uninfected cells were fixed and prepared as mock controls. For mScarlet analysis, cells were washed twice with DPBS and transferred to flow cytometry cluster tubes. For E antigen analysis, cells were permeabilized using 0.1% (vol/vol) saponin + 1% (vol/vol) FBS in DPBS for 15 min at room temperature. Cells were then stained with the orthoflavivirus group antigen antibody (clone 4G2) conjugated to Alexa Fluor 647 (Bio-Techne, Minneapolis, MN) and incubated for 1 h at 4 °C covered in foil. All information regarding antibodies used in this study can be found in Table S1. Cells were washed twice with DPBS, then transferred to flow cytometry cluster tubes. All samples were covered in foil until flow cytometry acquisition using the FACSymphony A3 with HTS Configuration (BD Biosciences, Franklin Lakes, NJ). The YG-610 detector was used to detect mScarlet-positive cells while the R-670 detector was used to detect E-positive cells. Results were analyzed using FlowJo v10 software (BD Biosciences, Franklin Lakes, NJ).

### Statistical analysis

Samples were analyzed as described in the figure legends. Statistical analysis was performed using GraphPad Prism v10.5.0. Multiple Welch *t* tests using the Holm–Sidak method were used to compare quantitative data between mScarlet(−) and mScarlet(+) sorted cell populations (*P < 0.05, **P < 0.01). Multiple Mann–Whitney tests using the Holm–Sidak method were used to compare quantitative data between mock and infected mice (*P < 0.05, **P < 0.01, ***P < 0.001).

A detailed description of all other methods can be found in the Supplementary material.

## Supplementary Material

pgag328_Supplementary_Data

## Data Availability

All data supporting the conclusions of this study are reported in the article. All raw data are publicly available on the Princeton University Data Commons website https://doi.org/10.34770/endy-0w24. All sequencing data (targeted NS1 viral sequencing, bulk RNA-seq of L929 cells, single-cell RNA-seq of splenocytes) have been deposited to NCBI GEO, under accession GSE312634. Reviewers may access this data using the password “ujwbeogafjedxgf”; these data will be made publicly available immediately following acceptance.
