# Supplementary material for: A versatile fluorescent reporter for Usutu virus drug screening and infection tracking
Source: PNAS Nexus. 2026 Sep 29;5(10):pgag328. doi: 10.1093/pnasnexus/pgag328 (PMC13659910; doi:10.1093/pnasnexus/pgag328)
Supplement: Supplementary file 1 [file pgag328_supplementary_data.zip › PNASNEXUS-PNASNEXUS-2025-01894-TRR-s11.pdf]

PNAS nexus

Supplementary Information for
A versatile fluorescent reporter for Usutu virus drug screening and infection tracking

Amy N. Nelson[1], Aaron E. Lin[1], Saloni Sinha[2], Sydney J. Mullin[1], Jianche Liu[1,3], Alexandra G. Bell[1], Sebastian S. Carver[1], Jason Sethiadi[2], Jiayu Zhang[1], Brigitte L. Heller[1], Cameron Myhrvold[1, 4–6], Robert E. Schwartz[2], Alexander Ploss[1, *]

[1] Department of Molecular Biology, Princeton University, Princeton, NJ 08544

[2] Division of Gastroenterology and Hepatology, Department of Medicine, Weill Cornell Medicine, New York, NY 10065

[3] Computational and Systems Immunology track, Stanford Immunology Program, Stanford University School of Medicine, Stanford, CA 94305

[4] Omenn-Darling Bioengineering Institute, Princeton University, Princeton, NJ 08544

[5] Department of Chemical and Biological Engineering, Princeton University, Princeton, NJ 08544

[6] Department of Chemistry, Princeton University, Princeton, NJ 08544

* Corresponding author: Alexander Ploss (A.P.)

Email: aploss@princeton.edu

This PDF file includes:

Supplementary text
Figures S1 to S8
Tables S1 to S4

Other supplementary materials for this manuscript include the following:

Datasets S1 to S2

Supplementary Information Text

Extended Materials and Methods

Antiviral drug screening using USUV-mScarlet

Huh7 cells were seeded in a 48-well plate at a density of 9.6 × 10^4 cells/well. The following day, cells were inoculated with USUV-mScarlet at an MOI of 0.01 in 0.1 mL of unsupplemented DMEM containing high glucose and pyruvate. Following incubation for 2 h at 37℃, the inoculum was

aspirated and replaced with 0.5 mL/well of DMEM containing high glucose and pyruvate supplemented with 10% (vol/vol) FBS and 1% (vol/vol) P/S and containing drug treatment. Tested concentrations of sofosbuvir (Selleck Chemicals, Houston, TX), ribavirin (MP Biomedicals, Irvine, CA), remdesivir (MedChemExpress, Monmouth Junction, NJ), and JNJ-A07 (MedChemExpress) ranged from 0.02–50 uM (3-fold dilutions) while tested concentrations of IFN-α2a (Sigma-Aldrich, St. Louis, MO) ranged from 5.12 × 10^{-5}–500 units (5-fold dilutions). The 50 uM dilutions of sofosbuvir, ribavirin, remdesivir, and JNJ-A07 were prepared in supplemented DMEM, while subsequent dilutions were prepared in supplemented DMEM + 0.1% (vol/vol) DMSO. All concentrations of IFN-α2a were prepared in supplemented DMEM without DMSO. Mock cells were maintained in supplemented DMEM or supplemented DMEM + 0.1% DMSO and were as negative controls or vehicle controls, respectively. Infected cells were harvested for flow cytometry staining at 48 hpi and analyzed for the frequency of mScarlet-positive cells following the methods described above. Samples were collected in quadruplicate over two independent experiments. Separately, the cytotoxicity of each drug concentration was tested in uninfected Huh7 cells using the Nano-Glo Luciferase Assay System (Promega, Madison, WI) according to the manufacturer’s protocol.

USUV-mScarlet passaging in cell culture to evaluate insert stability

Huh7 cells were seeded in a 6-well plate at a density of 9.6 × 10^5 cells/well. The following day, cells were inoculated with USUV-mScarlet at an MOI of 0.01 prepared in 1 mL of unsupplemented DMEM containing high glucose and pyruvate. Following incubation for 2 h at 37℃, the inoculum was aspirated and replaced with 2.5 mL/well of DMEM containing high glucose and pyruvate supplemented with 10% (vol/vol) FBS and 1% (vol/vol) P/S. 72 hours later, two 1 mL aliquots of cell culture supernatant were collected from each well and centrifuged at 5,000 rpm for 5 min at 4℃ to remove any cellular debris. 1 mL of supernatant was added to naïve Huh7 cells in a 6-well plate. The other 1 mL of supernatant was stored at -80℃ for downstream sequencing analysis. Any remaining supernatant was aspirated from the well, cells were washed with DPBS, treated with 0.05% (vol/vol) trypsin in Hank’s Balanced Salt Solution (Thermo Fisher Scientific, Waltham, MA), then quenched with supplemented cell culture medium. Harvested cells were centrifuged at 5,000 rpm for 5 min at 4℃, washed once with DPBS, then split into two aliquots. The first aliquot was resuspended in viral RNA buffer (Zymo Research, Irvine, CA) then stored at -80℃ for downstream RNA extraction. The

second aliquot of cells was resuspended in 4% (vol/vol) PFA and further prepared for flow cytometry as described above. Three independent replicates of USUV-mScarlet were passaged 10 times. Three uninfected (mock) supernatants and cells were also passaged 10 times and collected as negative controls. For viral RNA extraction, samples were thawed on ice then extracted using the Quick-RNA Viral kit (Zymo Research, Irvine, CA) according to the manufacturer's protocol.

Sequencing of viral RNA upstream of NS1 following USUV-mScarlet passages

Extracted viral RNA samples were treated with Turbo DNase (Thermo Fisher Scientific, Waltham, MA), then purified using a 0.8x SPRI cleanup. Reverse transcription was performed using an antisense primer mix from a fixed position within NS1, which simultaneously added an Illumina adapter. Primer sequences can be found in **Table S3**. cDNA was purified using a 0.7X SPRI cleanup following first and second strand synthesis based on the BRB-Seq protocol (71). Tagmentation was performed using Tn5 transposase to insert the Illumina R2 sequence. Therefore, cDNAs containing the fixed position of NS1 and the nearby upstream sequence were amplified by 17 cycles of PCR with standard Illumina indexing primers annealing to the R1 and R2 adapters. cDNAs were quantified by the High Sensitivity D1000 ScreenTape assay (Agilent Technologies, Santa Clara, CA), pooled and loaded on a MiSeq Nano v2 flowcell for 151 cycles R1, 8 cycles i7, and 151 cycles R2. Raw sequencing reads were demultiplexed by Picard v2.25.6 (from within viral-core v.2.3.1) using Q20M1 mismatch tolerance. Read pairs were mapped to the reference USUV-mScarlet genome (derived from GenBank AY453411.1). Discordant mapped read pairs, or pairs where R1 did not start at the expected fixed RT primer annealing position (due to non-specific priming), were removed. For remaining reads, the position of each sequencing read was identified and the distance between the fixed RT primer annealing position (R1) and the start of R2 was quantified as "insert size." For intact USUV-mScarlet, the median insert size should be 233 base pairs. Thus, an insert size threshold of 800 bp was set and defined as "far reads," which would occur if a read started in E due to deletion of the mScarlet reporter sequence. The percentage of far reads was quantified for each sample to indicate the amount of recombination that occurred.

Fluorescence-activated cell sorting (FACS) of mScarlet(+) and (–) populations

L929 mouse fibroblasts were infected with USUV-mScarlet (MOI: 1.0) and prepared for FACS at four time points: 12, 24, 36, and 48 hpi. For the 12 and 24 hpi time points, cells were seeded in 15 cm plates. For the 36 and 48 hpi time points, cells were seeded in 6 cm plates. For each time point, mock cells were seeded in 6-well plates. USUV-mScarlet inoculum was prepared in serum-free DMEM to achieve an MOI of 1.0 and incubated for 2 h at 37°C. At the appropriate time point, cells were trypsinized, quenched with supplemented DMEM, washed twice with 1% (vol/vol) FBS in DPBS, and transferred to 15 mL conical tubes. Cells were not fixed for FACS. Samples were run on a five-laser FACSAria Fusion cell sorter (BD Biosciences, Franklin Lakes, NJ). For each virally-exposed cell sample, 1×10^5 mScarlet(+) cells and 1×10^5 mScarlet(–) cells were collected. For each mock sample, 1×10^5 mScarlet(–) cells were collected. Cells were sorted into 1.5 mL microcentrifuge tubes and stored on ice. Cells were centrifuged at 5,000 rpm for 5 min at 4℃, then resuspended in RNA lysis buffer (Zymo Research, Irvine, CA). Cellular RNA was extracted using the Quick-RNA Miniprep Plus kit (Zymo Research, Irvine, CA) according to the manufacturer’s protocol. Extracted RNA was quantified by NanoDrop and Agilent 2100 BioAnalyzer (Agilent Technologies, Santa Clara, CA).

Library preparation and bulk RNA-sequencing

Samples were prepared by 3’ mRNA SMART-seq library preparation. Total RNA (300 ng) was used as input for reverse transcription using barcoded oligo-dT primers to label each sample in the 96-well plate following the drop-seq method (72). The pooled barcoded cDNA samples were amplified by PCR and purified, then turned into sequencing libraries using the Tn5 transpose-based tagmentation method to include only the poly-A tail adjacent 3′ ends of transcripts. Libraries were sequenced on one flowcell of a NovaSeq SP v1.5 flowcell (Illumina, San Diego, CA) with 112 cycles Read 1, 8 cycles Read i7, 8 cycles Read i5, and 10 cycles Read 2.

Bulk RNAseq analysis

Reads were demultiplexed with Picard v2.25.6 (from within viral-core v.2.3.1) using Q20M1 mismatch tolerance and the read structure flag ‘112T16S8B2S’ to process the within-pool sample barcode (from the RT primer). Next, reads were mapped to the mouse genome mm39, plus the USUV/TC508-mScarlet isolate reference (derived from GenBank AY453411.1), with STAR v2.7.11b, with uniquely mapping reads counted to the comprehensive gene annotation on the primary assembly with htseq-

count v2.0.5. Transcript read counts for mock, mScarlet(+) or mScarlet(–) cells at 12, 24, 36, and 48 hours post-infection (hpi) were analyzed using DESeq2 (v1.38.3). Biological replicates were first assessed for quality by calculating Pearson's correlation coefficients (PCC) of read counts across genes. Any replicate displaying a PCC < 0.9 compared to the other replicates was removed before DEG analysis. Low-abundance genes were filtered out by retaining only those with at least 10 reads in at least 2 samples. Differential expression analyses were conducted using raw read counts, as recommended by DESeq2, to compare the mScarlet(+), mScarlet(–), and mock cell populations at each time point. A gene was considered significantly differentially expressed if it had an adjusted p-value < 0.05 and an absolute log2 fold change > 2. Heatmaps were generated with the pheatmap (v1.0.12) package, using the Z-score of each gene for visualization. The 55 genes in the intersection between interferon-stimulated genes (ISGs) and significantly upregulated genes in mScarlet(+) or mScarlet(–) cells compared with mock cells at 48 hpi were selected and visualized in a heatmap. For Gene Set Enrichment Analysis (GSEA) analysis, differential genes identified were ranked according to their log2 fold-change values between mScarlet(+) or mScarlet(–) and mock cells at each time point. Ranked gene lists were subjected to using the clusterProfiler R package (v4.6.2) with the msigdbr database (v7.5.1) to access the Hallmark gene sets (category "H") curated for Mus musculus. GSEA was performed with an adjusted p-value cutoff of 0.05 and the normalized enrichment score (NES) was extracted for each pathway. The top 10 significantly enriched Hallmark gene sets from the 36 hpi comparison were selected, and these sets were used to interrogate gene set enrichment across all time points.

Mouse strains

Mice deficient for type I IFN receptor (Ifnar-/-) on a C57BL/6 genetic background were kindly provided by Dr. Sergei Kotenko at Rutgers New Jersey Medical School and generated as described previously (73). Stat1-/- mice were generated as previously described (34). Stat1[fl/fl] mice were kindly provided by Dr. Lothar Hennighausen (National Institute of Health, Bethesda, MD) and backcrossed for 10 generations to the C57BL/6 background as previously described (65). All mice were bred in the Laboratory Animal Resource (LAR) Center of Princeton University.

Mouse infections and monitoring experiments

Adult (6- to 14-week-old) male and female mice were infected through subcutaneous injection via nape of the neck with doses ranging from 10^3–10^6 FFU of USUV-mScarlet or 10^3 FFU of USUV-WT diluted in DPBS to a final volume of 200 µL. Signs of disease progression were recorded through weight measurements and clinical scoring daily. Overall appearance was assessed using a clinical scoring matrix assigned as follows: 0, posture normal, appearance with smooth, shiny fur; 1, posture hunched, appearance with ruffled fur, loss of muscle tone, loss of weight; 2, posture hunched, trembling, shaky, loss of weight; 3, posture severely hunched, disheveled appearance, significant (greater than or equal to 20%) weight loss, buildup of white residue around the eyes; 4, death. Mice with weight loss greater than or equal to 20% were euthanized by CO_2 chamber and their deaths were recorded for the following day.

Serum and tissue collection

Blood (ca. 200 µL) was collected through submandibular bleeding. Serum was separated from blood cells by centrifugation (3,500 rpm, 10 min, room temperature) and stored at -80°C in aliquots of 25 µL for later quantification of viremia. At the indicated endpoints, mice were anesthetized with ketamine/xylazine injection, and subject to cardiac puncture for a terminal blood collection. Spleen, liver, kidney, lung, and brain were harvested from mock or infected mice. Tissue sections were placed in 500 µL of RNAlater stabilization solution (Thermo Fisher Scientific, Waltham, MA) and stored at -80°C for downstream molecular analysis including RNA extraction and RT-qPCR. Tissue sections were placed in 3 mL of 10% neutral buffered formalin (MilliporeSigma) for 72 h at 4°C, then washed with DPBS and stored at 4°C in 5 mL of 70% (vol/vol) ethanol for downstream histological analysis.

RNA extraction from mouse serum and tissue

Tissue sections stored in RNAlater were thawed on ice. 10 mg of tissue was resuspended in 200 µL of MagMAX lysis/binding solution concentrate (Thermo Fisher Scientific, Waltham, MA) in a 2 mL microcentrifuge tube. A single 5-mm stainless steel bead (QIAGEN, Hilden, Germany) was placed into each tube containing tissue. Homogenization was accomplished using a TissueLyser LT (QIAGEN) set to 50 pulses per min for 2 min. After two rounds of homogenization separated by 2-min incubation on ice, the tubes were centrifuged at high speed (10,000 rpm) for 10 min at 4°C. The resulting supernatant was transferred to a 1.5-mL tube and used for viral RNA extraction. Viral RNA

was isolated from 25 µL of serum or 10 mg of homogenized tissue using the MagMax mirVana Total RNA Isolation Kit (Thermo Fisher Scientific, Waltham, MA) and the KingFisher Flex machine (Thermo Fisher Scientific, Waltham, MA) according to manufacturer's instructions. For each organ, two cuts (each 10 mg) were processed as biological replicates. For each sample, 30 µL of eluate was transferred to 0.2-mL strip tubes (USA Scientific, Ocala, FL) and subjected to DNase I treatment with Turbo DNase (Thermo Fisher Scientific, Waltham, MA). Lastly, viral RNA was purified by a 0.8x SPRI reaction using RNAClean XP beads (Beckman Coulter, Brea, CA). Purified RNA was stored in 0.2 mL strip tubes at -80°C until downstream RT-qPCR analysis.

Viral RNA quantification by reverse transcription-quantitative polymerase chain reaction (RT-qPCR)

Viral RNA quantification of cell culture lysates and supernatants was performed as previously described (34). Viral RNA quantification of mouse tissues was performed using the Luna Universal Probe One-Step RT-qPCR Kit (New England BioLabs Inc., Ipswich, MA) according to the manufacturer's protocol. Primers PU-O-13897 (TGAGAAGAAGCTCGGAGAGTTTGG) and PU-O-13898 (CCAATGGTCCTCATTCAGAAAGCC) and probe PU-O-13899 (AAGCGAAAGGTAGTCGAGCCATATGG) (Express PrimeTime 5' 6-FAM/ZEN/3' IB FQ probe, Integrated DNA Technologies, Morrisville, NC) were designed complementary to a region in USUV TC508 NS5. For each reaction 2 µL of RNA was used as sample input. The TOPO plasmid containing USUV CPER fragment 6 was used as the qPCR standard with a stock concentration of 7.32×10^9 DNA copies/µL. Nine 10-fold serial dilutions were prepared from the undiluted standard to create a standard curve from which cDNA copies in each sample were determined. RT-qPCR amplification was performed using a StepOnePlus Real Time PCR System (Thermo Fisher Scientific, Waltham, MA) with the following settings: 55°C for 10 min, 95°C for 1 min, then 40 cycles of 95°C for 10 s and 60°C for 30 s.

Histological analysis

Freshly harvested mouse tissues were fixed in 4% (w/v) paraformaldehyde for 24 h at 4°C, followed by dehydration and paraffin embedding. Tissue processing and sectioning were performed by Saffron Scientific Histology Services, LLC (Illinois, USA). Paraffin blocks were sectioned at 5 µm thickness

using a microtome, and sections were stained with hematoxylin and eosin (H&E) following standard procedures. Stained slides were imaged using a Zeiss Axioscan 7 slide scanner.

Tissue immunostaining

Paraffin-embedded tissue sections were deparaffinized in xylene and rehydrated through graded alcohols to deionized water. Antigen retrieval was performed by steaming in citrate buffer (pH 6.0) at 95°C for 20 min, followed by cooling to room temperature. Sections were blocked with 10% donkey serum in DPBS for 1 h at room temperature and subsequently incubated overnight at 4°C with primary antibodies, including anti-JEV-NS3 (GeneTex, Irvine, CA), anti-rabbit RFP pre-adsorbed (Rockland, PA). After PBS washes, sections were incubated with Alexa Fluor 488- or Alexa Fluor 546-conjugated donkey anti-rabbit IgG (H+L), highly cross-adsorbed secondary antibodies (Thermo Scientific, Waltham, MA), for 1 h at room temperature. Antibody dilutions and working concentrations are detailed in Table 1. Nuclei were counterstained with 4′,6-diamidino-2-phenylindole (DAPI; Thermo Scientific, Waltham, MA). Fluorescent images were acquired using a Zeiss LSM 880 confocal laser scanning microscope.

Preparation of mouse splenocytes for cell sorting

Stat1$^{-/-}$ mice were infected with 10^3 FFU of USUV-mScarlet. At 3 dpi, mice were anesthetized with ketamine/xylazine injection and their spleens were collected into a 15 mL conical tube containing 3 mL of DMEM with high glucose and pyruvate. Spleens were transferred into 6 cm tissue culture dishes, placed on steel mesh screens, and mechanically dissociated using the piston of a 3 mL syringe. Splenocytes were resuspended in 5 mL of digestion buffer composed of 0.1% (w/v) collagenase, 40 mM HEPES, 2 mM $CaCl_2$, 2 U/ml DNase I (Thermo Fisher Scientific, Waltham, MA) in Hanks' balanced salt solution (Thermo Fisher Scientific, Waltham, MA) for 15 min at 37°C. Digestion was quenched using 5 mL of DMEM containing high glucose and pyruvate + 10% (vol/vol) FBS + 1% (vol/vol) P/S. Cells were transferred to a 15 mL conical tube and centrifuged at 1,300 rpm at 4℃ for 5 min. The cell pellet was resuspended in 1X red blood cell lysis buffer in sterile H_2O (BD Biosciences, Franklin Lakes, NJ) and incubated for 15 min at room temperature covered in foil. Red blood cell lysis was quenched using 5 mL of supplemented DMEM and the cell suspension was passed through a 100-μm strainer. Splenocytes were washed twice with 1% (v/v) FBS in DPBS, then

cells were counted using 0.4% trypan blue solution (Thermo Fisher Scientific, Waltham, MA). Cells were stained using the Zombie UV fixable viability kit (BioLegend, San Diego, CA) diluted 1:100 in DPBS and incubated for 15 min at room temperature covered in foil. A live/dead control containing 1×10^6 splenocytes was prepared using 50% live splenocytes and 50% dead splenocytes that were heat-killed at 95℃ for 10 min. Cells were washed using 1% (v/v) FBS in DPBS. Cells were stained using anti-mouse CD45 conjugated to FITC (**Table S1**) diluted 1:400 in 1% (v/v) FBS in DPBS, then washed twice using 1% (v/v) FBS in DPBS. Samples were run on a five-laser FACSAria Fusion cell sorter (BD Biosciences, Franklin Lakes, NJ).

Single-cell RNA sequencing of sorted mouse splenocytes

For each sample, we prepared aliquots of either 80,000 sorted cells, or 80,000 sorted cells plus 4,200 mouse hepatoma Hepa1-6 cells (5% spike-in control). We performed cell hashing (74) with 0.25 µg of each TotalSeq anti-mouse Hashtag Antibodies 1–8 (BioLegend, San Diego, CA), ultimately superloading the Chromium Controller (10X Genomics, Pleasanton, CA) with a pool of 33,000 cells to recover 20,000 cells across all samples, per lane in duplicate lanes. We prepared libraries with the Chromium Next GEM Single Cell 3' Kit v3.1 (10X Genomics), using 11 PCR cycles for cDNA and hashtag oligo amplification. For gene expression libraries, we recovered ~150 ng cDNA per lane and carried over 25% (~38 ng) for remaining library prep including 13 cycles of sample index PCR. For hashtag oligo libraries, we performed 21 cycles of feature barcode PCR. We pooled hashtag oligo libraries (5%) and gene expression libraries (95%) and sequenced on a NovaSeq SP v1.5 flowcell (Illumina) with 28 cycles Read 1, 8 cycles Read i7, and 101 cycles Read 2.

scRNA-seq analysis

Raw sequencing reads were processed with CellRanger v7.2 (10X Genomics). Reads were demultiplexed with the ‘mkfastq’ command, and then cell calling, read assignment, and read mapping to the mouse mm10 genome supplemented with the reference USUV-mScarlet genome (derived from GenBank AY453411.1), was performed with the ‘multi’ command. Samples were aggregated into a counts matrix with the ‘aggr’ command for further analysis. Counts data was analyzed with scanpy v1.11.5 (75). We removed cells with few genes or counts, or too high mitochondrial content. Cells were clustered with the Leiden algorithm (76), and then assigned broad cell types based on known

marker genes (B: *Ebf1*, T: *Cd3d*, myeloid: *Itgam* + *Ifitm2*). Clusters containing fewer than 100 cells, potential doublets, or without clear lineage identity (clusters 23–28) were excluded from further analysis. Within these broad types, we performed differential gene expression analysis (Wilcoxon rank-sum test) to identify the top 50 ranked genes associated with each cluster for further manual annotation of cell subtypes.

Figures

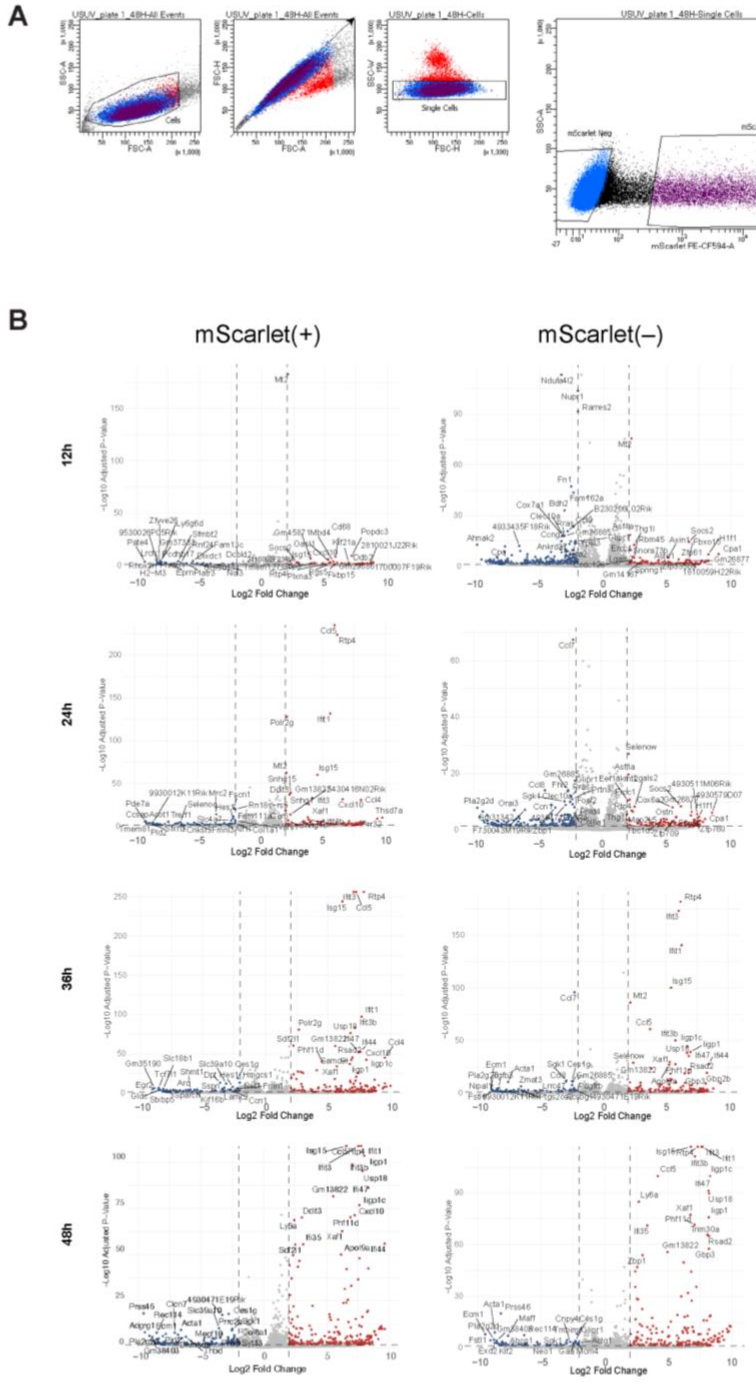

Fig. S1. Fluorescence-activated cell sorting of mScarlet(+) and mScarlet(–) L929 cell populations for bulk RNA-seq. (*A*) Gating scheme used to collect mScarlet(+) and mScarlet(–) populations at 48 hours post-infection (hpi). First, a gate was drawn to separate cells from debris based on side scatter (SSC-A) vs. forward scatter (FSC-A). Then, a gate was drawn to select single cell events from doublets based on FSC-H vs. FSC-A and SSC-W vs. FSC-H. Lastly, distinct gates were drawn to

collect mScarlet$^+$ or mScarlet$^-$ cells based on SSC-A vs. mScarlet signal. (*B*) Volcano plots of differentially expressed genes (DEGs) in L929 cells exposed to USUV-mScarlet (MOI: 1.0) at 12, 24, 36, and 48 hpi. Red dots represent up-regulated genes, blue dots represent down-regulated genes, and gray dots represent insignificant genes.

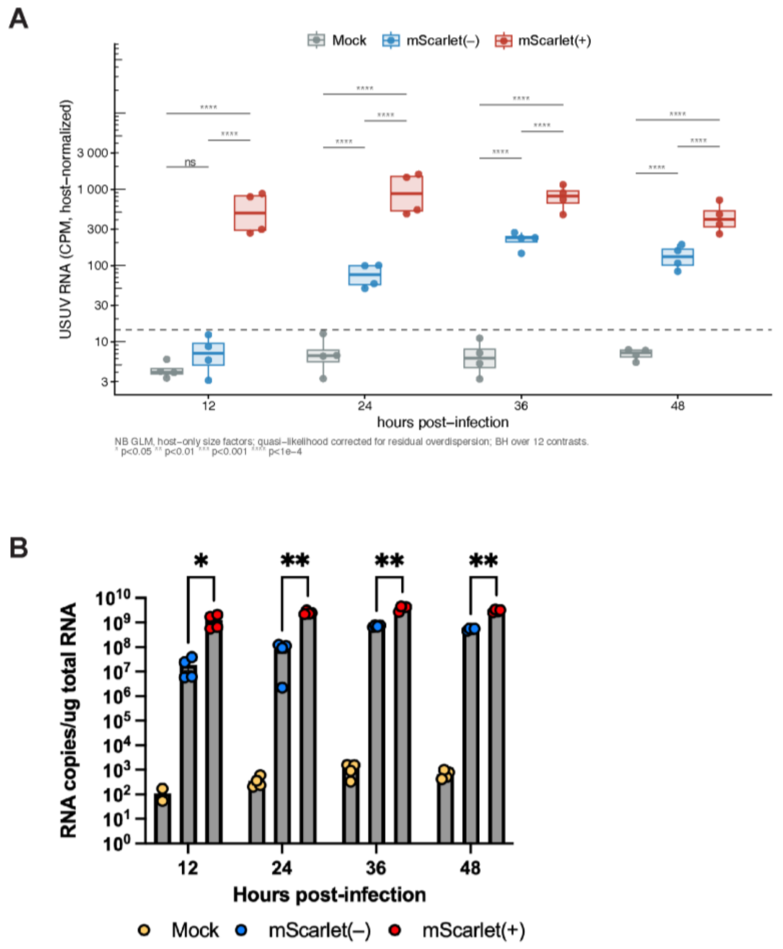

Fig. S2. Assessment of USUV reads in sorted L929 cell populations. (*A*) Viral reads per million host reads in mock, mScarlet(–), and mScarlet(+) populations. Using DESeq2, a negative binomial GLM test was performed on raw counts, then USUV counts were converted to viral counts per million to normalize for varying library sizes. (*B*) Quantification of viral RNA in mock, mScarlet(–), and mScarlet(+) populations (n = 4/sample type) by RT-qPCR. Statistical analysis was performed using GraphPad Prism v10.5.0. Multiple Welch t tests using the Holm-Sidak method were used to compare quantitative data between mScarlet(–) and mScarlet(+) sorted cell populations (*P < 0.05, **P < 0.01).

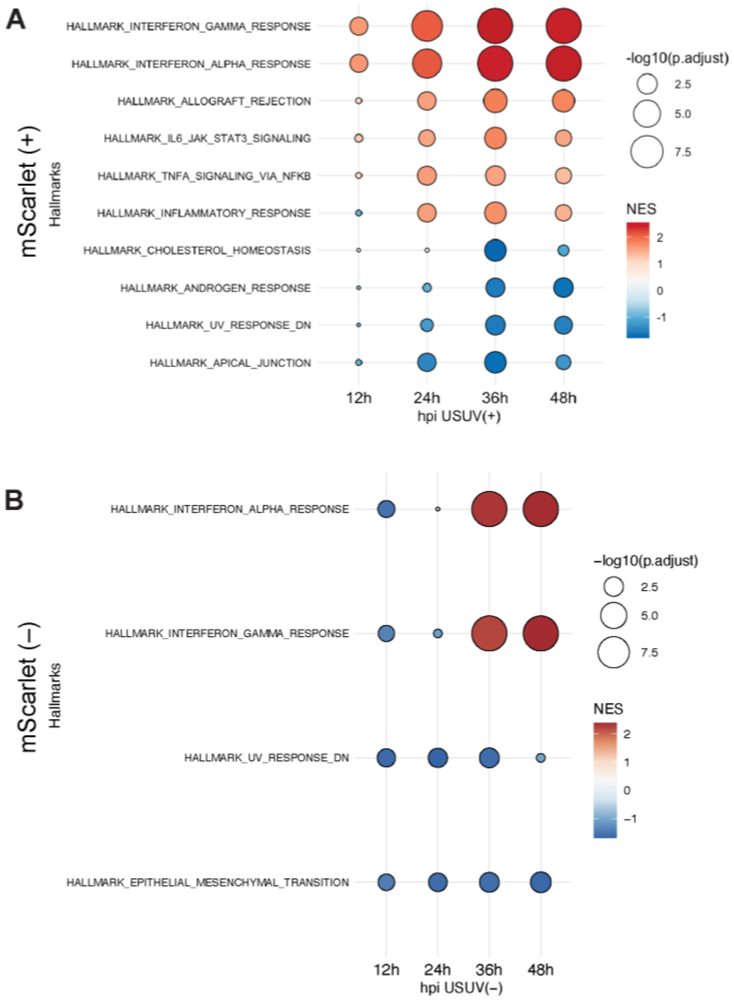

Fig. S3. Gene set enrichment analyses demonstrate the importance of the type I IFN response in mScarlet(+) and mScarlet(–) L929 mouse fibroblasts. (*A*) The top 10 significantly enriched hallmark gene sets in the mScarlet(+) sorted populations of L929 cells at 12, 24, 36, and 48 hpi. (*B*) The top 4 significantly enriched hallmark gene sets in the mScarlet(–) sorted populations of L929 cells at 12, 24, 36, and 48 hpi. GSEA was performed with an adjusted p-value cutoff of 0.05 and the normalized enrichment score (NES) was extracted for each pathway.

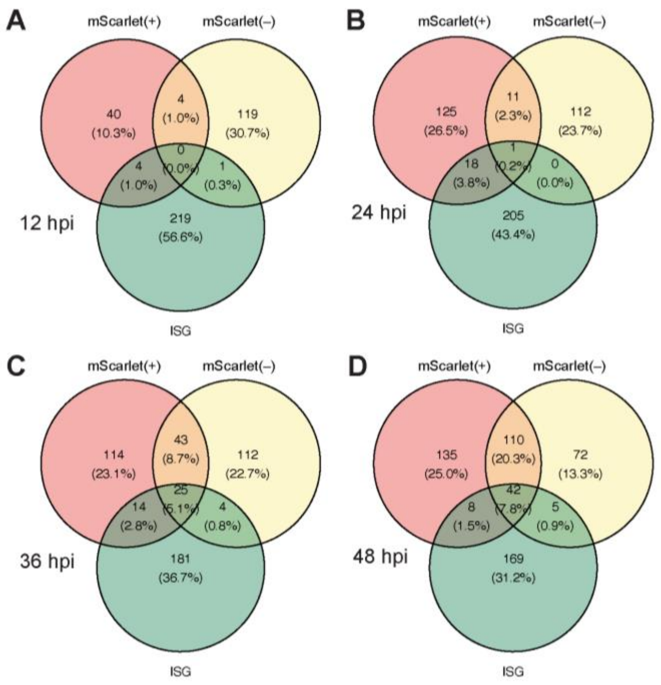

Fig. S4. Similar transcriptomic profiles marked by upregulation of interferon-stimulated genes (ISGs) were observed in mScarlet(+) and mScarlet(–) L929 cells exposed to USUV-mScarlet (MOI: 1.0). The overlap of the quantity of all significantly upregulated genes mScarlet(+) cells (shown in red) and mScarlet(–)cells (shown in yellow) and the quantity of ISGs (green) expressed by L929 cells at (A) 12 hpi, (B) 24 hpi, (C) 36 hpi, and (D) 48 hpi.

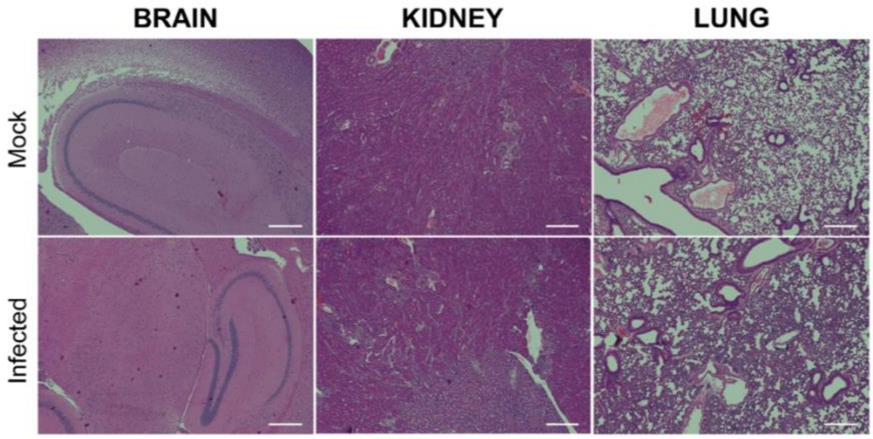

Fig. S5. Histological analysis of kidney, brain, and lung collected from Ifnar-/- mice infected with USUV-mScarlet at 6 dpi. Representative sections of H&E staining performed on brain, kidney, and lung harvested from mock- or infected mice. Infected mice were subcutaneously inoculated with 10^3 FFU of USUV-mScarlet. Scale bars indicate 200 μm.

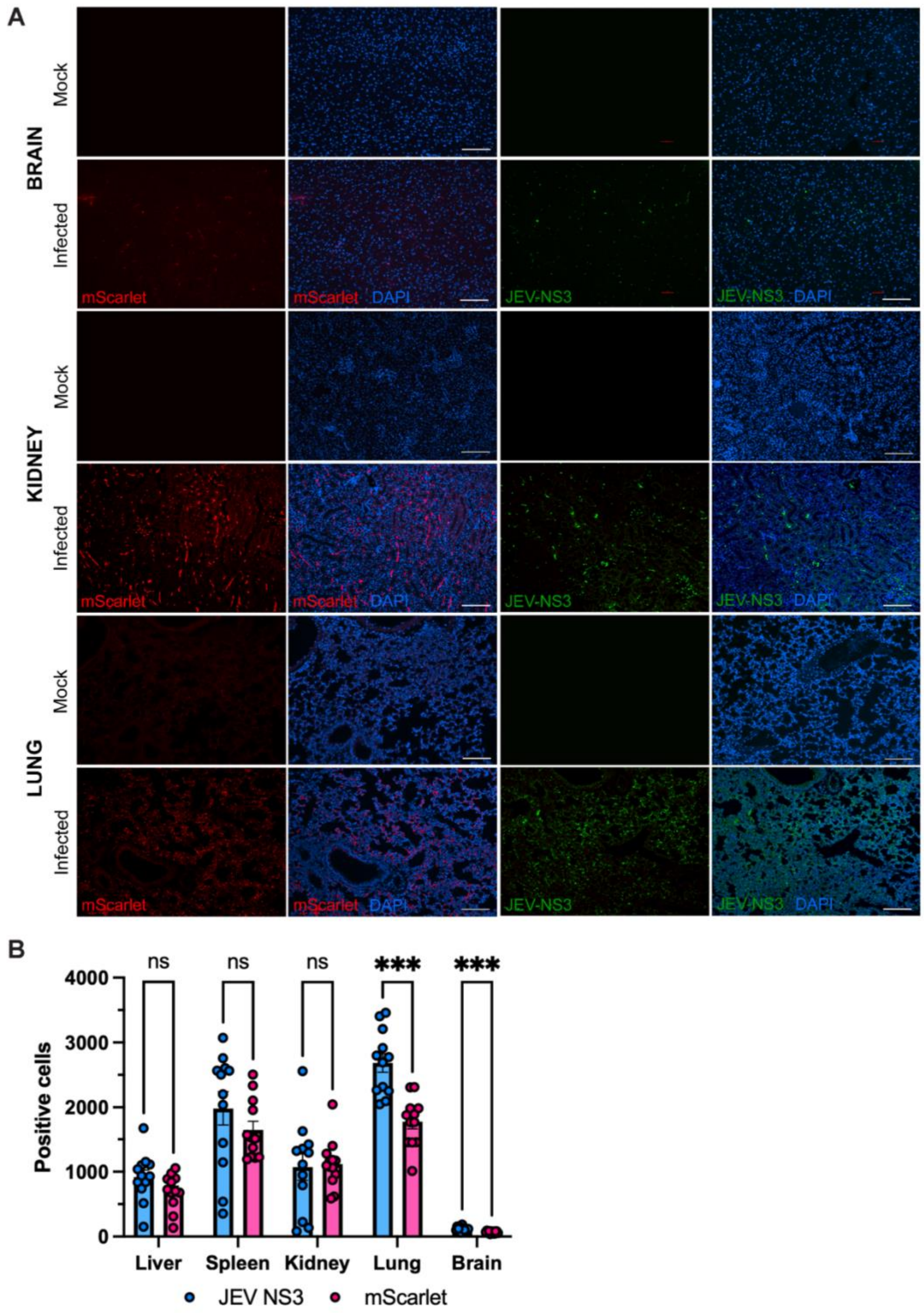

Fig. S6. Immunostaining of kidney, brain, and lung collected from Ifnar-/- mice infected with USUV-mScarlet at 6 dpi. (A) Representative sections of brain, kidney, and lung harvested from mock- or USUV-mScarlet-infected mice (10^3 FFU). Immunostaining for mScarlet and JEV NS3 proteins confirms viral dissemination to and replication within these tissues. Nuclei were counterstained with DAPI. Scale bars indicate 200 µm. (B) Quantification of JEV NS3+ cells compared to mScarlet+ cells in liver, spleen, kidney, lung, and brain tissue sections harvested from Ifnar1-/- mice at 6 dpi. For each

tissue, three sections—taken at different z-axis depths—were stained and quantified. All mock tissues showed complete absence of JEV NS3+ or mScarlet+ cells and were not plotted. Mann-Whitney tests using the Holm-Sidak method were used to compare the frequencies of JEV $NS3^+$ and $mScarlet^+$ cells (*P < 0.05, **P < 0.01, ***P < 0.001).

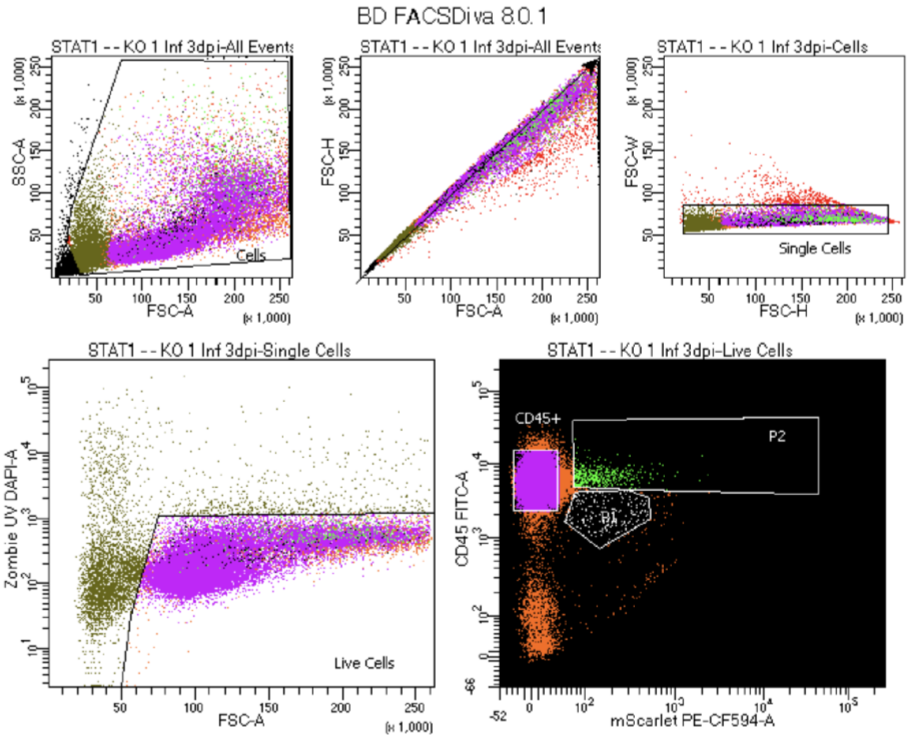

Tube: STAT1 - - KO 1 Inf 3dpi

Population	#Events	%Parent	%Total
All Events	44,574	####	100.0
Cells	38,823	87.1	87.1
Single Cells	38,245	98.5	85.8
Live Cells	33,687	88.1	75.6
CD45+	29,360	87.2	65.9
P1	220	0.7	0.5
P2	460	1.4	1.0

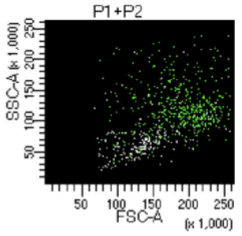

Experiment Name: AN_20240209_USUV Spleen for scRNA Seq
Tube Name: STAT1 - - KO 1 Inf 3dpi
Record Date: Feb 9, 2024 1:35:26 PM

Population	#Events	%Parent	CD45 FITC-A Median	mScarlet PE-CF594-A Median
All Events	44,574	####	5,344	11
Cells	38,823	87.098	5,309	10
Single Cells	38,245	98.511	5,286	10
Live Cells	33,687	88.082	5,491	9
CD45+	29,360	87.155	5,608	8
P1	220	0.653	1,950	165
P2	460	1.366	6,696	126

Fig. S7. Gating scheme for fluorescence-activated cell sorting of CD45$^+$mScarlet$^-$ and CD45$^+$mScarlet$^+$ splenocyte populations from a Stat1-/- mouse for scRNA sequencing. First, all cells were gated separate from red blood cell debris. Next, single cells were selected based on FSC-H vs. FSC-A and FSC-W vs. FSC-H, thereby excluding doublets. Next, live cells were selected based on negative staining for Zombie UV viability dye. Lastly, infected and non-infected immune cells were separated based on CD45 and mScarlet signals. The CD45$^+$mScarlet$^-$ population is shown in purple and the CD45$^+$mScarlet$^+$ population is shown in green.

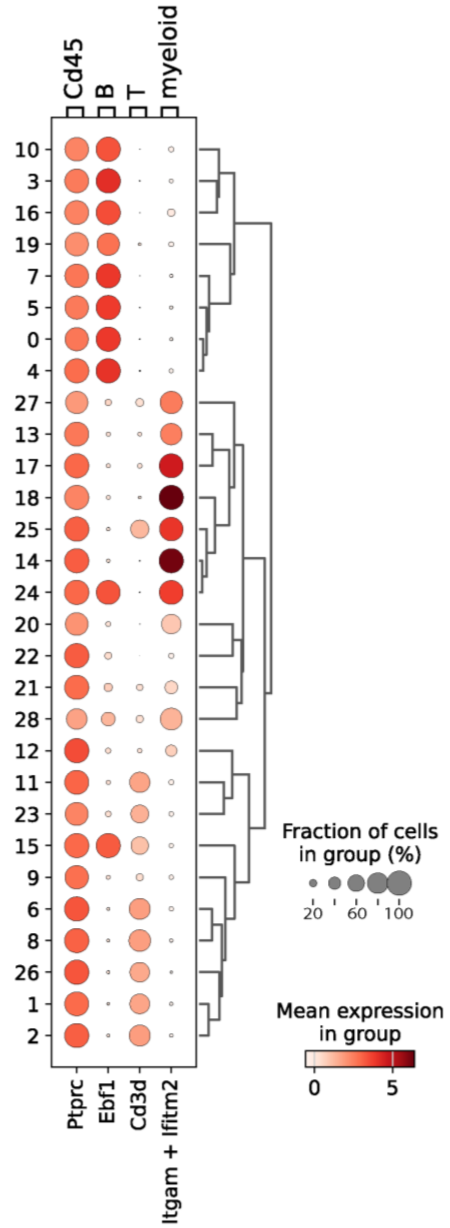

Fig. S8. Dot plot used for classification of scRNAseq clusters as B lymphocytes, T lymphocytes, or myeloid cells. Classification as a B cell was based on *Ebf1* expression, classification as a T cell was based on *Cd3d* expression and classification as a myeloid cell was based on combined *Itgam* + *Ifitm2* expression.

Tables

Table S1. Antibodies used in this study for flow cytometry or histology staining.

Antibody and stock concentration	Manufacturer	Identifier	Dilution	Final concentration
Anti-mouse Flavivirus group antigen (4G2) conjugated to Alexa Fluor 647 (1 mg/mL)	Bio-Techne	NBP2-52709AF647	1:100	10 µg/mL
Anti-rabbit Japanese encephalitis virus NS3 (1 mg/mL)	GeneTex	GTX125868	1:100	10 µg/mL
Anti-rabbit RFP antibody pre-absorbed (1 mg/mL)	Rockland Immunochemicals	600-401-379	1:100	10 µg/mL
Donkey anti-rabbit IgG (H+L) Highly Cross-Adsorbed Secondary Antibody, Alexa Fluor 488 (2 mg/mL)	Thermo Fisher Scientific	A-21206	1:400	5 µg/mL
Donkey anti-rabbit IgG (H+L) Highly Cross-Adsorbed Secondary Antibody, Alexa Fluor 546 (2 mg/mL)	Thermo Fisher Scientific	A10040	1:400	5 µg/mL
Anti-mouse CD45 monoclonal antibody (30–F11), FITC (0.5 mg/mL)	BioLegend	103108	1:400	1.25 µg/mL

Table S2. Primers for inserting mScarlet into the USUV TOPO F2 plasmid for infectious clone generation.

Fragment component	Primer names	F primer sequence (5' to 3')	R primer sequence (5' to 3')	Plasmid template
Overlapping extension PCR piece 1	F: PU-O-11167<br>R: PU-O-11330	CAGCGTCTAG CTGCCCTAGG CGACACAGCG TGGGACTTTG	GCCGACATCGA TAGCGCAGCCG GAATCTGCATG GACGTTTGTGG C	USUV CPER Fragment 2 TOPO
Overlapping extension PCR piece 2	F: PU-O-11331<br>R: PU-O-11170	CGGCTGCGCT ATCGATGTCG GCGGCGCCAT GGTGAGC	CGCTTGAACCAT GACTCCTAG	pACNR-FLYF-17D-mScarlet-I
Overlapping extension PCR piece 3	F: PU-O-11171<br>R: PU-O-11172	GTCATGGTTC AAGCGGACTC GGGATGTGCG ATAGAC	GCCCGTCAACC ACAAAAGTGTG GTTGGCCAGTT CTG	USUV CPER Fragment 2 TOPO

Table S3. Reverse transcription primers for sequencing USUV-mScarlet passages.

Primer name	Primer sequence (5’ to 3’)
PU-O-13768	TCCCTACACGACGCTCTTCCGATCTNNNNNNAATCCCTTGTCCACAGCG
PU-O-13769	TCCCTACACGACGCTCTTCCGATCTNNNNNNNAATCCCTTGTCCACAGCG
PU-O-13770	TCCCTACACGACGCTCTTCCGATCTNNNNNNNNAATCCCTTGTCCACAGCG
PU-O-13771	TCCCTACACGACGCTCTTCCGATCTNNNNNNNNNAATCCCTTGTCCACAGCG
PU-O-13772	TCCCTACACGACGCTCTTCCGATCTNNNNNNNNNNAATCCCTTGTCCACAGCG

Cluster #	Identity	Top transcripts
0	Follicular/naive B cells	Bach2, Cd55, Ighd, H2-Aa, Fcer2a, Cr2
1	Naive CD4 T cells	Cd4, Bach2, Lef1, Tcf7
2	Naive CD8 T cells	Cd8b1, Cd8a, Lef1, Themis
3	Activated B cells	Cr2, Plac8, Cd86
4	Follicular/naive B cells	Fcer2a, Fcmr, Bach2, Ighd
5	Germinal center B cells	Cd74, Bcl6, H2-Ab1, Ighd, Pik3ap1
6	CD8+ effector T cells (NK-like)	Ccl5, Ly6c2, Il2rb, Il18r1, Nkg7, Gramd3, Klrd1
7	Immature/Naive B cells	Ighm, Vpreb3, Bach2, Cd79a/b
8	CD8+ effector T cells (NK-like), cycling cells	Nkg7, Il2rb, Npm1, Eif5a, Furin
9	CD8+ effector T cells (NK-like)	Gm26917, Nr4a3, Klf6, Dusp2, Cd69, Gramd3, Itk, Themis
10	Marginal Zone B cells	Fcrl5, Ighm, Sox5, Plac8
11	Activated regulatory T cells	Ctla4, Ikzf2, Maf, Rora, Tox, S100a6, Cd44
12	NK cells	Nkg7, Gzma, Il2rb, Klrb1c (NK1.1), Ncr1
13	Type 2 conventional dendritic cells (cDC2s)	Ctss, Ctsz, Cd74, Cd86, H2-Aa, H2-Ab1, H2-Eb1
14	Monocytes, early inflammatory	Cst3r, Il1b, Il1r2, Acod1, Cxcl2
15	Activated T cells	Skap1, Itk, Prkch, Themis, Cd3d, Cd28, Cd247
16	Transitional/recently activated B cells	Sox4, Nr4a3, Egr1, Pik3r4
17	Monocyte-derived macrophages, IFN-responsive	Lyz2, Cst3, Ccl6, Csf1r, Plac8
18	Neutrophils	Ngp, S100a8, S100a9, Lcn2, Camp
19	Proliferating plasmablasts	Iglc1, Xbp1, Jchain, Mki67, Top2a, Stmn1, Kif11
20	Migratory (activated) DCs (conventional DCs)	Cd74, Cd83, Flt3, H2-Aa, H2-Eb1
21	Spleen resident red pulp macrophages	Vcam1, Hmox1, C1qa, C1qb, C1qc
22	pDCs	Siglech, Bst2, Tcf4, Ccr9

Table S4. Identification of all Leiden clusters from scRNA sequencing analysis based on their top 50 differentially expressed transcripts. B lineage cells are highlighted in blue, T lineage cells are highlighted in green, and myeloid lineage cells are highlighted in pink. Cluster 23–28 contained less than 100 cells and could not be reliably identified. Top 50 transcript lists for all clusters can be found in **Dataset S2**.

Other supplementary materials for this manuscript:

Dataset S1 (separate file). ISG expression profiles for mScarlet(+) and mScarlet(–) L929 cells at 12, 24,36, and 48 hpi with USUV-mScarlet.

Dataset S2 (separate file). Top 50 transcripts expressed by each scRNAseq cluster.
